# 14‐3‐3 protein inhibits CaMKK1 by blocking the kinase active site with its last two C‐terminal helices

**DOI:** 10.1002/pro.4805

**Published:** 2023-10-20

**Authors:** Olivia Petrvalska, Karolina Honzejkova, Nicola Koupilova, Petr Herman, Veronika Obsilova, Tomas Obsil

**Affiliations:** ^1^ Department of Physical and Macromolecular Chemistry, Faculty of Science Charles University Prague Czech Republic; ^2^ Institute of Physiology of the Czech Academy of Sciences, Laboratory of Structural Biology of Signaling Proteins Division BIOCEV Vestec Czech Republic; ^3^ Institute of Physics, Faculty of Mathematics and Physics Charles University Prague Czech Republic

**Keywords:** 14‐3‐3 proteins, calcium/calmodulin‐dependent protein kinase, CaMKK, fluorescence spectroscopy, hydrogen/deuterium exchange coupled to MS, protein–protein interaction, SAXS

## Abstract

Ca^2+^/CaM‐dependent protein kinase kinases 1 and 2 (CaMKK1 and CaMKK2) phosphorylate and enhance the catalytic activity of downstream kinases CaMKI, CaMKIV, and protein kinase B. Accordingly, CaMKK1 and CaMKK2 regulate key physiological and pathological processes, such as tumorigenesis, neuronal morphogenesis, synaptic plasticity, transcription factor activation, and cellular energy homeostasis, and promote cell survival. Both CaMKKs are partly inhibited by phosphorylation, which in turn triggers adaptor and scaffolding protein 14‐3‐3 binding. However, 14‐3‐3 binding only significantly affects CaMKK1 function. CaMKK2 activity remains almost unchanged after complex formation for reasons still unclear. Here, we aim at structurally characterizing CaMKK1:14‐3‐3 and CaMKK2:14‐3‐3 complexes by SAXS, H/D exchange coupled to MS, and fluorescence spectroscopy. The results revealed that complex formation suppresses the interaction of both phosphorylated CaMKKs with Ca^2+^/CaM and affects the structure of their kinase domains and autoinhibitory segments. But these effects are much stronger on CaMKK1 than on CaMKK2 because the CaMKK1:14‐3‐3γ complex has a more compact and rigid structure in which the active site of the kinase domain directly interacts with the last two C‐terminal helices of the 14‐3‐3γ protein, thereby inhibiting CaMKK1. In contrast, the CaMKK2:14‐3‐3 complex has a looser and more flexible structure, so 14‐3‐3 binding only negligibly affects the catalytic activity of CaMKK2. Therefore, Ca^2+^/CaM binding suppression and the interaction of the kinase active site of CaMKK1 with the last two C‐terminal helices of 14‐3‐3γ protein provide the structural basis for 14‐3‐3‐mediated CaMKK1 inhibition.

## 1 INTRODUCTION

**FIGURE 1:**
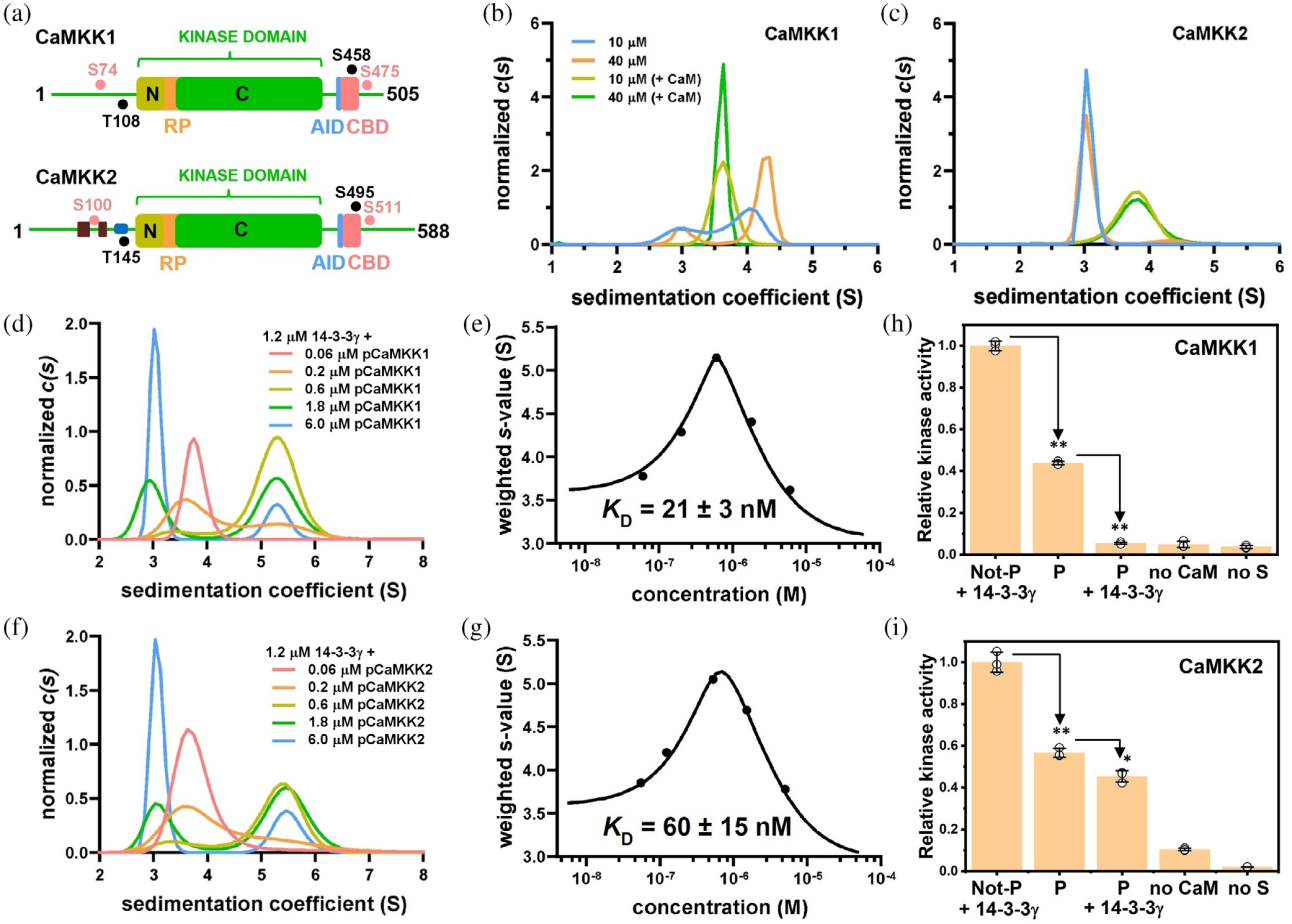
pCaMKKs interact with 14‐3‐3γ with high binding affinity through two binding motifs. (a) Domain structure of human CaMKK1 and CaMKK2. AID, autoinhibitory domain; CBD, Ca^2+^/CaM‐binding domain. The positions of the four PKA phosphorylation sites are indicated by dots (the 14‐3‐3 binding motifs are indicated in pink). The dark blue bar indicates the regulatory 23‐amino acid stretch of CaMKK2 (residues 129–151). Insertions into the CaMKK2 sequence are indicated by brown bars. (b, c) Comparison of area‐normalized *c*(*s*) distributions of CaMKK1 (b) and CaMKK2 (c) at two different concentrations with and without Ca^2+^/CaM (1:1 molar ratio). (d) Series of area‐normalized *c*(*s*) distributions of mixtures of pCaMKK1 and 14‐3‐3γ at various molar ratios, using 1.2 μM 14‐3‐3γ and 0.06–6 μM pCaMKK1. (e) Binding curve of weight‐average sedimentation coefficients *s*_w_ calculated from SV AUC experiments with mixtures of 14‐3‐3γ and pCaMKK1. (f) Series of area‐normalized *c*(*s*) distributions of mixtures of pCaMKK2 and 14‐3‐3γ at various molar ratios, using 1.2 μM 14‐3‐3γ and 0.06–6 μM pCaMKK2. (g) Binding curve of weight‐average sedimentation coefficients *s*_w_ calculated from SV AUC experiments with mixtures of 14‐3‐3γ and pCaMKK2. (h, i) Differences in 14‐3‐3‐mediated (h) CaMKK1 and (i) CaMKK2 inhibition. The catalytic activity of CaMKK1 and CaMKK2 was measured using human CaMK1D (kinase‐dead mutant D165A) as a specific substrate. The activities were normalized to the non‐phosphorylated enzyme activity (specific activities were 4300 ± 200 and 2440 ± 60 nmol min^−1^ mg^−1^ for CaMKK1 and CaMKK2, respectively). The results are expressed as mean ± SD, *n* = 3. Not‐P, non‐phosphorylated enzyme; P, phosphorylated enzyme; no CaM, no Ca^2+^/CaM; no S, no substrate; *, *p* < 0.05; **, *p* < 0.005.

One of the main calcium signaling mechanisms is based on the interaction of calmodulin, a calcium‐sensing protein, with Ca^2+^/CaM‐dependent protein kinases (CaMKs) (reviewed in Clapham, 2007; Marcelo et al., 2016). All CaMKs share a similar domain structure with a kinase domain followed by a regulatory region containing an autoinhibitory segment (AID) and a binding region for the Ca^2+^/CaM complex (CBD) (Figure 1a). In the resting state, kinase activity is blocked by AID, which prevents substrate binding and/or affects the structure of the active center. When the calcium concentration increases, a Ca^2+^/CaM complex is formed, which binds to the regulatory region and disrupts its interaction with the kinase domain, thus preventing autoinhibition. The activation of some CaMKs requires not only Ca^2+^/CaM binding but also phosphorylation, either by autophosphorylation or by other protein kinases, such as Ca^2+^/CaM‐dependent protein kinase kinases 1 and 2 (CaMKK1 and CaMKK2) (Anderson et al., 1998; Tokumitsu et al., 1995; Tokumitsu and Sakagami, 2022).

CaMKKs phosphorylate and enhance the catalytic activity of downstream kinases CaMKI, CaMKIV, and protein kinase B (PKB/Akt), thereby regulating key physiological and pathological processes. These processes include tumorigenesis, neuronal morphogenesis, synaptic plasticity, transcription factor activation, and cell survival (Mizuno et al., 2006; Peters et al., 2003; Yano et al., 1998; Gocher et al., 2017; Massie et al., 2011). Furthermore, CaMKK2 phosphorylates and activates the catalytic subunit of 5′ adenosine monophosphate‐activated protein kinase (AMPK), a major regulator of cellular energy homeostasis (Anderson et al., 2008; Hurley et al., 2005). For these reasons, both CaMKKs are considered promising targets for therapeutic intervention, including treating different types of cancer and promoting weight loss (Price et al., 2018; Eduful et al., 2021).

Despite their high sequence homology and similar domain structure (Figures 1a and S1), CaMKK1 and CaMKK2 differ in the regulation of their catalytic activities. CaMKK1 is regulated in a strictly Ca^2+^/CaM‐dependent manner, whereas CaMKK2 shows considerable CaM‐independent activity. This activity is regulated by a 23‐amino‐acid stretch (residues 129–151) located N‐terminally to the catalytic domain (Green et al., 2011; Tokumitsu et al., 2001). Both CaMKKs are phosphorylated at multiple sites (Ser^52^, Ser^74^, Thr^108^, Ser^458^, and Ser^475^ in CaMKK1; Ser^100^, Thr^145^, Ser^495^, and Ser^511^ in CaMKK2) by cAMP‐dependent protein kinase (PKA) (Wayman et al., 1997; Matsushita and Nairn, 1999; Langendorf et al., 2020; Takabatake et al., 2019). PKA‐mediated phosphorylation partly inhibits both kinases by modifying the Thr residue in the segment preceding the kinase domain (Thr^108^ in CaMKK1 and Thr^145^ in CaMKK2) and the Ser residue located in CBD (Ser^458^ in CaMKK1 and Ser^495^ in CaMKK2), whose phosphorylation reduces their Ca^2+^/CaM binding affinity (Wayman et al., 1997).

In addition to partial inhibition, CaMKK phosphorylation by PKA exposes binding motifs for adaptor and scaffolding proteins 14‐3‐3. 14‐3‐3 proteins are abundant dimeric proteins that regulate the function of hundreds of other proteins in a phosphorylation‐dependent manner by recognizing motifs containing a phosphorylated Ser or Thr residue (reviewed in Obsilova and Obsil, 2022). 14‐3‐3 proteins simultaneously bind to two phosphorylated motifs of both CaMKKs, one located at the N‐terminus, upstream of the kinase domain, and the other at the C‐terminus, just downstream of the regulatory region (Figure 1a). The C‐terminal motif (containing Ser^475^ in CaMKK1 and Ser^511^ in CaMKK2) binds to 14‐3‐3 proteins with higher affinity and most likely functions as the “gatekeeper”, that is, the dominant motif that ensures the overall stability of the complex (Langendorf et al., 2020; Yaffe, 2002; Lentini Santo et al., 2020) Although 14‐3‐3 proteins bind to the N‐terminal motif (Ser^74^ in CaMKK1 and Ser^100^ in CaMKK2) with lower affinity, this bond ensures the biological function of the interaction and further stabilizes the complex. Thus, 14‐3‐3 protein binding further modulates the catalytic activity of CaMKK1 and 2.

Functional in vitro and in vivo studies have suggested that 14‐3‐3 binding protects pSer of the CBD of both CaMKKs from dephosphorylation, indicating that 14‐3‐3 binding maintains CaMKKs in a PKA‐mediated partly inhibited state. However, the remaining catalytic activity of CaMKK1 and CaMKK2 is differently affected by this interaction. CaMKK1 is strongly inhibited by 14‐3‐3 binding, whereas CaMKK2 activity remains almost unchanged, but the underlying mechanism(s) remain(s) unclear (Langendorf et al., 2020; Ichimura et al., 2008; Davare et al., 2004; Psenakova et al., 2018).

To understand differences in the 14‐3‐3‐mediated regulation of human CaMKK1 and CaMKK2, we performed a biophysical and structural characterization of both complexes using an integrated approach based on analytical ultracentrifugation, hydrogen/deuterium exchange coupled to MS (HDX–MS), fluorescence spectroscopy, and small‐angle X‐ray scattering (SAXS). Our results show that both CaMKKs have the same domain arrangement and bind to 14‐3‐3γ with similar high affinity, but their interactions with 14‐3‐3γ differ considerably, thus providing a structural basis for their differences in 14‐3‐3 protein‐mediated regulation.

## 2 RESULTS

### 2.1 Both CaMKKs interact with 14‐3‐3γ with a similarly high binding affinity

CaMKKs contain two 14‐3‐3 binding motifs flanking the kinase domain (containing Ser^74^ and Ser^475^ in CaMKK1; Ser^100^ and Ser^511^ in CaMKK2) (Figures 1a and S1). Their simultaneous phosphorylation and interaction with 14‐3‐3 proteins enhance both the overall stability of the complexes and the inhibitory effect of the interaction (Langendorf et al., 2020; Ichimura et al., 2008; Psenakova et al., 2018). Therefore, structural analysis was performed with catalytically inactive CaMKKs constructs containing both 14‐3‐3 binding motifs, the kinase domain and the AID and CBD regions (CaMKK1_67–480_ D293A and CaMKK2_93–517_ D330A, hereafter referred to as CaMKK1 and CaMKK2).

Because PKA phosphorylates the N‐terminal 14‐3‐3 binding motif much more quickly than the C‐terminal motif, the previously published phosphorylation protocol was optimized to ensure stoichiometric phosphorylation of both sites (Psenakova et al., 2018). Our LC–MS analysis confirmed stoichiometric phosphorylation not only of both 14‐3‐3 binding motifs but also of two additional PKA sites in the CaMKK constructs (Thr^108^ and Ser^458^ in CaMKK1; Thr^145^ and Ser^495^ in CaMKK2) (Figures S2 and S3).

CaMKKs oligomerization in solution was characterized by sedimentation velocity analytical ultracentrifugation (SV‐AUC). These experiments revealed that unphosphorylated CaMKK1, but not CaMKK2, dimerizes in solution in a concentration‐dependent manner. The sedimentation coefficient distribution *c*(*s*) of CaMKK1 at 10 μM concentration showed two peaks with weight‐average sedimentation coefficients corrected to 20.0°C and to the density of water, *s*_w(20,w)_, of 2.88 ± 0.01 and 4.14 ± 0.04 S with a frictional ratio *f*/*f*_0_ of 1.32 (estimated *M*_w_ ~ 39 and ~58 kDa, respectively), most likely corresponding to the CaMKK1 protomer and dimer (Table S1 and Figure 1b). In contrast, the sedimentation coefficient distribution *c*(*s*) of CaMKK2 showed only one peak with a *s*_w(20,w)_ of 3.2 ± 0.03 S with a frictional ratio *f*/*f*_0_ of 1.35 (estimated *M*_w_ ∼ 42 kDa) (Table S1 and Figures 1c and S4b). Both CaMKKs formed a stable complex with Ca^2+^/CaM, as evidenced by the shift of the peak to higher *s* values (Table S1 and Figure 1b,c). The *c*(*s*) distributions of the CaMKK1:Ca^2+^/CaM and CaMKK2:Ca^2+^/CaM complexes showed *s*_w(20,w)_ values of 3.8 ± 0.03 S, with a frictional ratio *f*/*f*_0_ of 1.43 (estimated *M*_w_ ∼ 58 kDa), and 3.9 ± 0.04 S, with a frictional ratio *f/f*_0_ of 1.36 (estimated *M*_w_ ∼ 57 kDa), respectively, indicating the formation of complexes with 1:1 molar stoichiometry. CaMKK1 dimerization was partly attenuated by PKA phosphorylation and completely blocked by Ca^2+^/CaM binding (Figures 1b and S4). Based on these results, the Ca^2+^/CaM binding domain (CBD) of CaMKK1 containing one of the four PKA phosphorylation sites (Ser^458^, Figure 1a) may be involved in its dimerization.

CaMKKs interact with several human 14‐3‐3 protein isoforms (Langendorf et al., 2020; Ichimura et al., 2008; Davare et al., 2004; Psenakova et al., 2018). Because the 14‐3‐3γ isoform often shows higher binding affinity to 14‐3‐3 binding partners than to other isoforms (Gogl et al., 2021), 14‐3‐3γ was used throughout this study. The interaction between phosphorylated CaMKKs (denoted as pCaMKK1 and pCaMKK2) and 14‐3‐3γ was characterized by SV‐AUC analysis of their mixtures at various molar ratios (0.06–6 μM pCaMKKs and 1.2 μM 14‐3‐3γ) (Figure 1d–g). Based on the sedimentation coefficient distribution *c*(*s*), 14‐3‐3γ and pCaMKK1 and pCaMKK2 formed complexes with *s*_w(20,w)_ of 5.57 ± 0.05 S with a frictional ratio *f*/*f*_0_ of 1.33 and 5.78 ± 0.02 S with a frictional ratio *f*/*f*_0_ of 1.36, respectively. These *s*_w(20,w)_ values correspond to molecular weights of ~93 and ~102 kDa, respectively, suggesting a 2:1M stoichiometry for both complexes (the theoretical molecular weights are 101.2 and 102.2 kDa, respectively). Direct modeling of the SV AUC data using the Lamm equation showed best‐fit apparent equilibrium dissociation constants (*K*_D_) of 21 ± 3 and 60 ± 15 nM using a Langmuir binding model, with pCaMKK1 and pCaMKK2 molecules interacting with a 14‐3‐3γ homodimer.

### 2.2 Differences in 14‐3‐3γ‐mediated pCaMKK1 and pCaMKK2 inhibition

Previous studies have shown that 14‐3‐3 binding differently affects the catalytic activity of pCaMKK1 and pCaMKK2 (Langendorf et al., 2020; Ichimura et al., 2008; Davare et al., 2004; Psenakova et al., 2018). Complex formation inhibits pCaMKK1, whereas pCaMKK2 activity remains minimally affected (Ichimura et al., 2008; Davare et al., 2004; Psenakova et al., 2018). This difference was confirmed by our kinase activity measurements with recombinant catalytically active CaMKK1_67–480_ and CaMKK2_93–517_ and human CaMK1D as specific substrate (Figure 1h,i). Phosphorylation by PKA reduced the kinase activity of CaMKK1 by approximately 56%, and the addition of 14‐3‐3γ completely inhibited the remaining activity, rendering pCaMKK1 catalytically inactive. Conversely, CaMKK2 activity was inhibited by PKA‐mediated phosphorylation by approximately 43%, and the presence of 14‐3‐3γ only slightly reduced the remaining activity. In addition, CaMKK2, but not CaMKK1, showed significant Ca^2+^/CaM‐independent activity. These results corroborate previous findings and show that the truncated CaMKK1 and CaMKK2 constructs that were used in this study mimicked WT proteins.

### 2.3 14‐3‐3γ binding affects the accessibility of the active site and autoinhibitory segment of pCaMKK1

**FIGURE 2:**
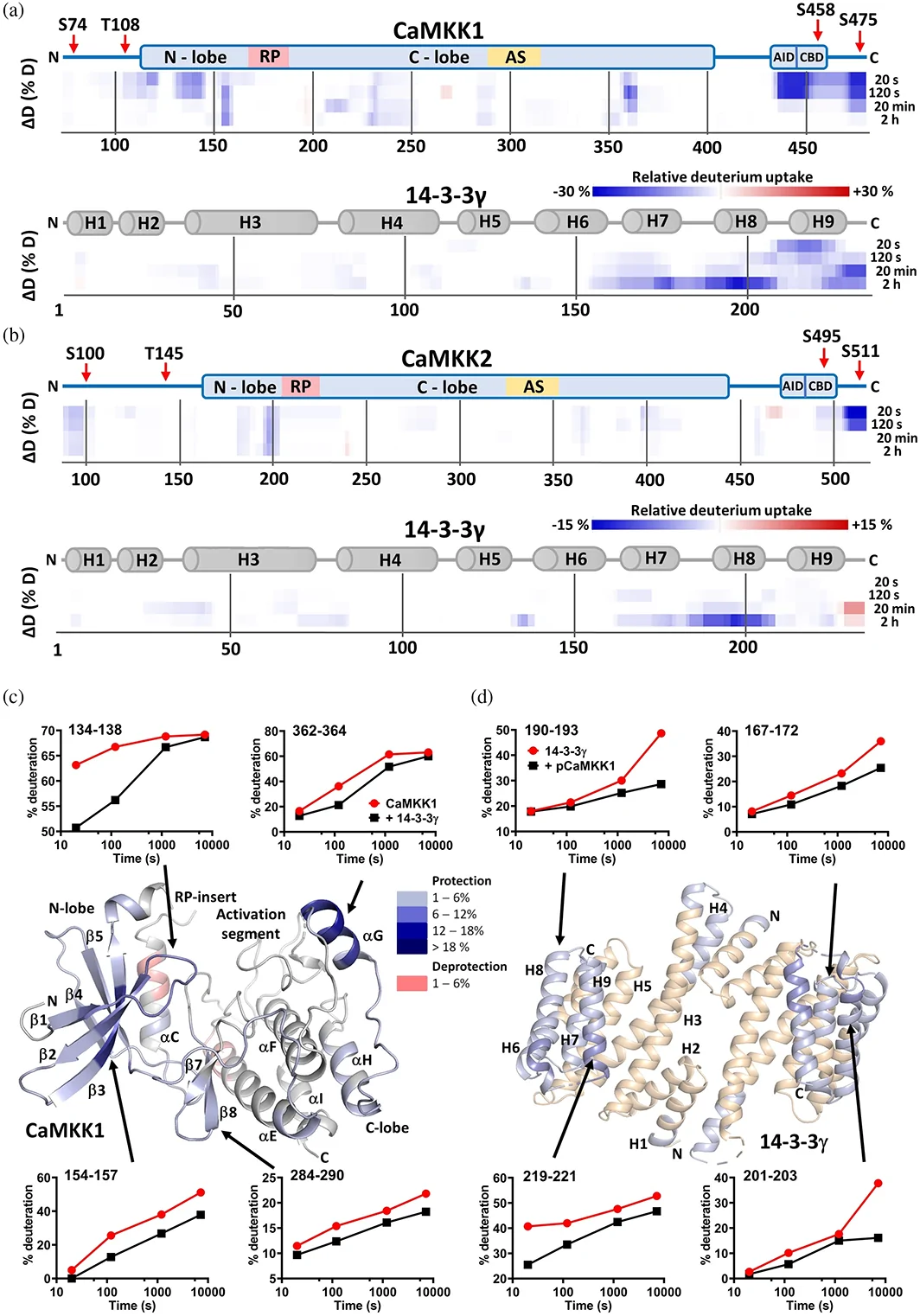
HDX–MS reveals the binding interface of pCaMKK:14‐3‐3γ complexes. (a, b) H/D heat maps showing differences in deuteration of pCaMKKs and 14‐3‐3γ after complex formation for 20 s, 120 s, 20 min, and 2 h of incubation. Blue and red indicate lower and higher deuteration uptake (from −30% to 30% for the pCaMKK1:14‐3‐3γ complex; from −15% to 15% for the pCaMKK2:14‐3‐3γ complex). The domain structure of CaMKKs and the secondary structure of 14‐3‐3γ are indicated at the top. The positions of PKA phosphorylation sites in CaMKKs are indicated by red arrows. (c, d) Crystal structures of the kinase domain of CaMKK1 (PDB ID: 6CD6) and 14‐3‐3γ (PDB ID: 6FEL (Psenakova et al., 2018)) with mapped changes in deuteration kinetics after complex formation for deuteration time 120 s. Graphs show representative HDX kinetics for selected pCaMKK1 and 14‐3‐3γ regions with slower deuterium exchange kinetics after complex formation. Deuterium exchange is expressed as percentage of the maximum theoretical deuteration level of pCaMKK1 or 14‐3‐3γ alone (red circles) and in a complex (black squares).

Since differences in 14‐3‐3‐mediated inhibition (Figure 1h,i) suggest different interactions between 14‐3‐3γ and CaMKK1 and 2, these differences were analyzed by investigating structural changes induced by complex formation by HDX–MS. This method is used to monitor the kinetics of hydrogen‐to‐deuterium exchange along the polypeptide backbone. Some hydrogens of the amide groups are protected from exchange thanks to their involvement in stable hydrogen bonds and/or to steric shielding from the solvent. Conversely, highly flexible regions exposed to the solvent show faster exchange than rigid and buried sections (Wales and Engen, 2006; Masson et al., 2019). The exchange kinetics of pCaMKK1, pCaMKK2, and 14‐3‐3γ regions were followed on 414, 282, and 273 peptides from pepsin digestion, together covering 99.5%, 99.3%, and 98.3% of the sequence, respectively (Figures S5–S7). When comparing pCaMKK1 deuteration profiles with and without 14‐3‐3γ, we found significant protection (lower levels of deuterium uptake) of several pCaMKK1 regions upon 14‐3‐3γ binding (Figures 2a,c and S8). The highest protection after 20‐s‐ and 2‐min‐long deuterations was observed in the C‐terminal region (residues 434–480), especially in the autoinhibitory segment and in the C‐terminal 14‐3‐3 binding motif containing pSer^475^. Furthermore, other regions with a considerably slower deuterium uptake in the presence of 14‐3‐3γ included the 111–121 region preceding the kinase domain, two regions from the N‐lobe of the kinase domain (the 131–146 region forming strands β1, β2 and the phosphate‐binding P‐loop located between them and the 154–157 region within the strand β3), and the 358–365 region containing the helix αG from the C‐lobe of the kinase domain. The β1–3 strands and the phosphate‐binding P‐loop form one side of the ATP‐binding pocket of the active site. However, deuteration plots for longer incubation times (20 min and 2 h) showed lower levels of protection of the whole enzyme, suggesting that longer conformational fluctuations of bound pCaMKK1 exposed amide hydrogens (Deredge et al., 2016). In addition, little protection was observed at the N‐terminus, where the N‐terminal 14‐3‐3 binding motif (pSer^74^) is located, even at short deuteration times.

The comparison of deuteration profiles of 14‐3‐3γ with and without pCaMKK1 revealed high protection, mainly within the last three C‐terminal helices H7–H9 (Figures 2a,d and S9). These helices form one side of the binding groove where the phosphorylated motif binds and the outside surfaces of helices H8 and H9 are also frequently involved in contacts with 14‐3‐3 protein binding partners (Obsilova and Obsil, 2022). These data indicate that pCaMKK1 interacts with these surfaces outside the central channel of the 14‐3‐3γ dimer. Moreover, in 14‐3‐3γ, the level of protection, especially in the H8 helix region, increases with deuteration time. This increase indicates that this region is stably involved in the interaction with pCaMKK1 and that 14‐3‐3γ is stabilized and/or less accessible upon complex formation.

In turn, HDX analysis of the pCaMKK2:14‐3‐3γ complex showed considerably less pronounced changes in the deuteration profiles of both pCaMKK2 and 14‐3‐3γ than in that of the pCaMKK1:14‐3‐3γ complex (Figures 2b, S10, and S11), suggesting a more solvent‐accessible and/or dynamic arrangement of the complex. Strong protection was identified only at short deuteration times and in the region 491–517 of pCaMKK2 containing the C‐terminal 14‐3‐3 binding motif (pSer^511^). In addition, weak protection was noted in the N‐terminus region 91–105, containing the N‐terminal 14‐3‐3 binding motif (pSer^100^), and in the region 197–204 of the N‐lobe of the kinase domain, which forms the β3 strand and the beginning of the RP‐insert. For 14‐3‐3γ proteins, protection was observed only in the H7 and H8 helix region and only at long deuteration times, as in the complex with pCaMKK1. Overall, our HDX–MS analysis showed that 14‐3‐3γ binding decreases solvent accessibility and/or exchange dynamics in several regions of the pCaMKK1 molecule, including the active site and the autoinhibitory segment, whereas the solvent accessibility of pCaMKK2 is less affected, most likely because the complex is less compact and/or more flexible.

### 2.4 Complex formation with 14‐3‐3γ suppresses Ca^2+^/CaM binding to CaMKKs

We have previously shown that the interaction with 14‐3‐3γ partly inhibits Ca^2+^/CaM binding to phosphorylated CaMKK2 and that this partial inhibition requires phosphorylation of the C‐terminal 14‐3‐3 binding motif (Psenakova et al., 2018). But when CaMKK2 was phosphorylated only at the N‐terminal motif, 14‐3‐3 binding had no effect on the interaction between pCaMKK2 and Ca^2+^/CaM. Nevertheless, the CaMKK2 used in our previous study was only partly phosphorylated at the C‐terminal 14‐3‐3 binding motif. To fully assess the effect of PKA‐mediated phosphorylation and 14‐3‐3γ binding on the ability of pCaMKKs to interact with Ca^2+^/CaM, we performed binding experiments with dansyl‐labeled CaM (DANS‐CaM) and both CaMKKs phosphorylated either only at their 14‐3‐3 binding sites (denoted as pCaMKK1‐2P and pCaMKK2‐2P) or at all four PKA sites, with and without 14‐3‐3γ.

**FIGURE 3:**
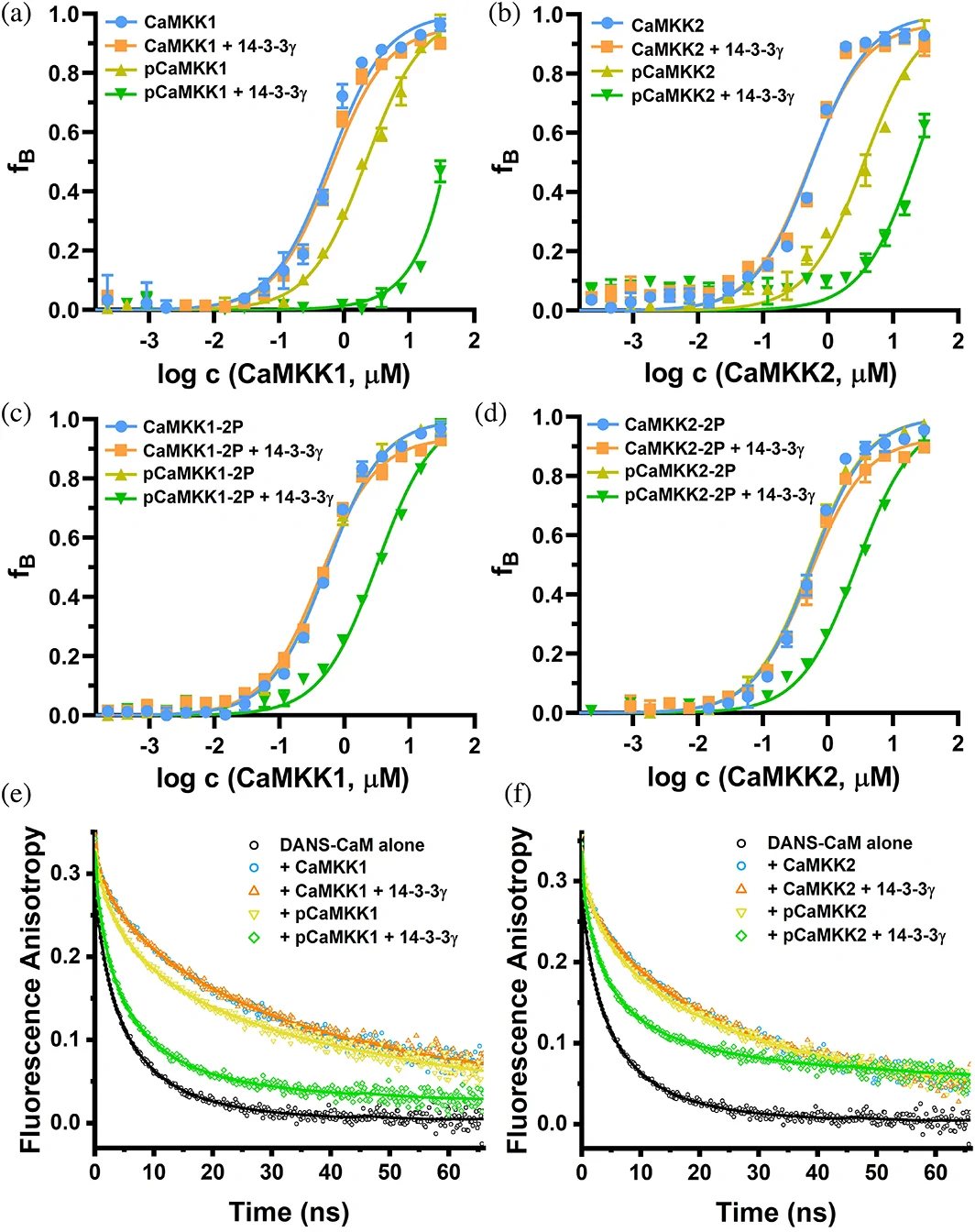
Interaction with 14‐3‐3γ suppresses Ca^2+^/CaM binding to CaMKKs. (a, b) Fluorescence polarization (FP) measurements of DANS‐labeled CaM with and without 14‐3‐3γ titrated by CaMKK1/2 or pCaMKK1/2. The binding affinities were determined by fitting the FP data to a one‐site‐binding model. (c, d) FP measurements of DANS‐labeled CaM with and without 14‐3‐3γ titrated by CaMKK1/2‐2P or pCaMKK1/2‐2P. All data points are the mean ± SD of three replicates. (e, f) Time‐resolved fluorescence anisotropy decays of free dansyl‐Ca^2+^/CaM and dansyl‐Ca^2+^/CaM with CaMKKs (or pCaMKKs) and with and without 14‐3‐3γ.

A fluorescence polarization (FP)‐based binding assay showed that phosphorylation alone, but especially subsequent 14‐3‐3γ binding, reduced the binding affinity of both CaMKKs to CaM (Figure 3a,b and Table S2) and that the inhibitory effect of 14‐3‐3γ binding was stronger on pCaMKK1 than on pCaMKK2. In unphosphorylated CaMKKs, however, 14‐3‐3γ had no effect on CaM binding. The same experiments with pCaMKKs‐2P confirmed that phosphorylation of the 14‐3‐3 binding motifs alone had no effect on CaM binding, so the reduced binding affinity to CaM in pCaMKKs results only from the phosphorylation of the Ser residue in their CBD (Wayman et al., 1997; Langendorf et al., 2020). Nevertheless, the interaction with 14‐3‐3γ significantly reduced the CaM binding affinity of both pCaMKKs‐2P (Figure 3c,d and Table S2).

CaM displacement from the complex with pCaMKKs was subsequently monitored by time‐resolved fluorescence anisotropy decay measurements of DANS‐CaM (Figure 3e,f). CaMKK:Ca^2+^/CaM complexes were clearly formed, as shown by the considerably slower DANS‐CaM fluorescence anisotropy decays due to an increase in the fraction of the complex with higher values of the longest correlation times *ϕ*_3_ and *ϕ*_4_ (Table S3) with the consequent decrease in the overall rotational diffusion of DANS‐CaM. In addition, the formation of the CaMKK1/2:Ca^2+^/CaM complexes also increased the mean excited‐state lifetime (*τ*_mean_) of the dansyl moiety, suggesting its shielding from the polar environment upon binding to CaMKKs.

PKA‐mediated phosphorylation of CaMKKs somewhat destabilized their interaction with DANS‐CaM, but 14‐3‐3γ binding to pCaMKKs caused DANS‐CaM dissociation from both kinases. This effect was also considerably stronger on pCaMKK1 than on pCaMKK2. DANS‐CaM dissociation associated with increased dansyl mobility is highlighted by the raw data (green diamonds in Figure 3e,f) and by an elevated free DANS‐CaM fraction. This increased fraction is reflected by the decrease in the values of the short correlation times *ϕ*_1_ and *ϕ*_2_, which closely resemble the values of free DANS‐CaM, and by the increased values of the associated amplitudes *β*_1_ and *β*_2_ (Table S3). As also shown by the raw data, 14‐3‐3γ had only a negligle effect on DANS‐CaM binding to unphosphorylated CaMKKs.

### 2.5 14‐3‐3 forms a more compact and less flexible complex with pCaMKK1 than with pCaMKK2

**FIGURE 4:**
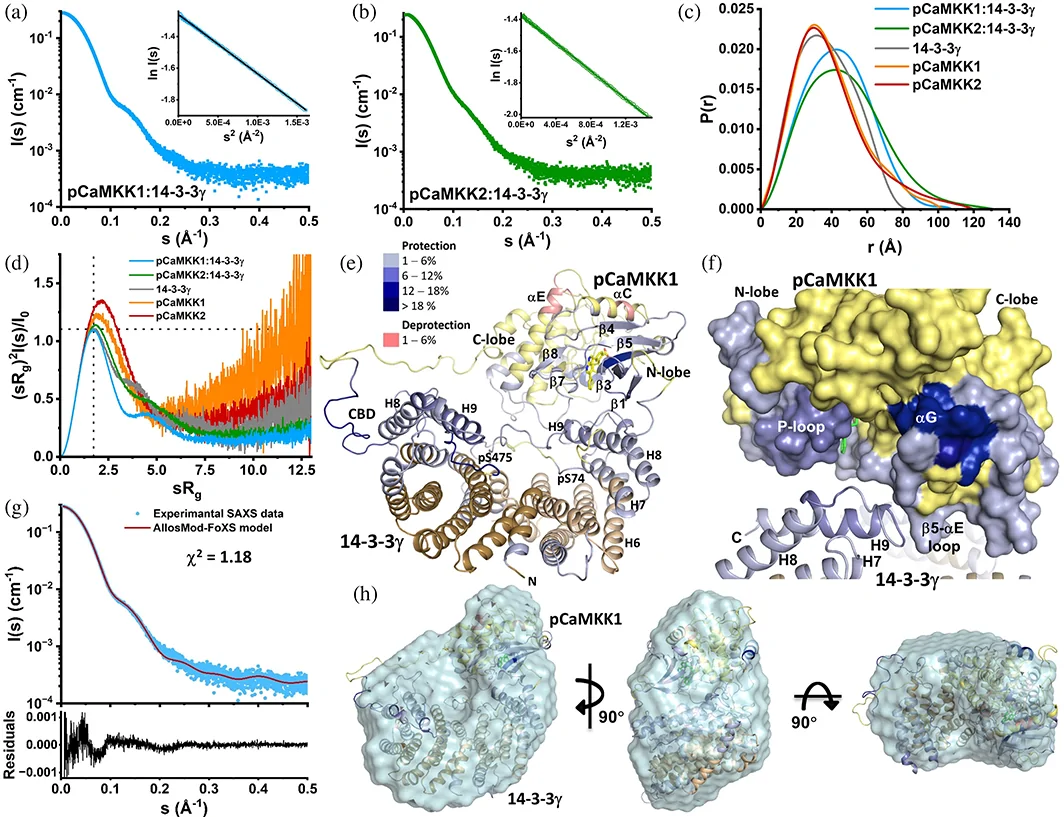
SAXS‐based structural analysis of the pCaMKK1:14‐3‐3γ complex. (a, b) Scattering intensity as a function of the momentum transfer *s* (*s* = 4*π*sin(*θ*/*λ*), where 2*θ* is the scattering angle, and *λ* is the wavelength) of the pCaMKK1:14‐3‐3γ and pCaMKK2:14‐3‐3γ complexes. The insets show the Guinier plot of the scattering data. (c) Distance distribution functions *P*(*r*) calculated from scattering data. (d) Dimensionless Kratky plots. Dotted lines mark the maximum at a value of 1.104 for *sR*_g_ = 1.73, which is typical of scattering data of compact globular proteins (Receveur‐Brechot and Durand, 2012). (e) Ribbon and surface representations of the best‐scoring AllosMod‐FoXS model of the pCaMKK1:14‐3‐3γ complex constructed using crystal structures of the kinase domain of CaMKK1 (PDB ID: 6CD6) and 14‐3‐3γ with bound CaMKK phosphopeptides (PDB ID: 6FEL and 6EWW (Psenakova et al., 2018)). The model is colored according to changes in deuteration kinetics after complex formation for a deuteration time of 120 s. The inhibitor present in the crystal structure of the kinase domain of CaMKK1 (yellow sticks) is shown to indicate the position of the active site. (f) Detailed view of the binding interface between the C‐terminal part of 14‐3‐3γ (shown as ribbon) and the kinase domain of pCaMKK1 (shown as surface, N‐ and C‐terminal segments are not shown). (g) Experimental scattering curve of the pCaMKK1:14‐3‐3γ complex superimposed with the calculated curve of the best‐scoring AllosMod‐FoXS model of the complex (shown in red). (h) Filtered molecular envelope of the pCaMKK1:14‐3‐3γ complex (pale cyan envelope) calculated from SAXS data with a superimposed AllosMod‐FoXS model of the complex.

Both pCaMKK:14‐3‐3γ complexes (prepared with 1:2 molar stoichiometry) and 14‐3‐3γ, CaMKK1, pCaMKK1, CaMKK2, and pCaMKK2 were characterized by size exclusion chromatography (SEC) coupled to small angle X‐ray scattering (Table S4 and Figures 4a,b, S12, and S13). Model‐independent analysis of SAXS data confirmed the formation of complexes with the expected stoichiometry (Table S4). Moreover, the apparent *M*_w_ values of CaMKK1 and CaMKK2 in the unphosphorylated and phosphorylated states and the 14‐3‐3γ dimer calculated from the SAXS data agree well with their expected *M*_w_ values based on their sequences.

The values of the gyration radius (*R*_g_) calculated using the Guinier approximation (Guinier plots are shown in insets, in Figures 4a,b and S13), and the GNOM program indicated that the pCaMKK1:14‐3‐3γ complex is more compact than the pCaMKK2:14‐3‐3γ complex (Table S4). Furthermore, the value of the volume‐of‐correlation (*V*_C_) (Rambo and Tainer, 2013) was smaller, also indicating a more compact pCaMKK1:14‐3‐3γ complex (Table S4 and Figure 4c). Considering these results, we compared the conformational flexibility of all proteins using dimensionless Kratky and Porod–Debye plots of scattering data (Figures 4d and S14). The dimensionless Kratky plots ((*sR*_g_)^2^ *I*(*s*)/*I*_0_ vs. *sR*_g_, where *s* is the momentum transfer, *I*(*s*) is the scattering intensity, and *I*_0_ is the extrapolated intensity at zero angle) revealed that the pCaMKK1:14‐3‐3γ complex is somewhat less conformationally heterogeneous than the pCaMKK2:14‐3‐3γ complex, as indicated by the position of the curve maxima (1.10 at *sR*_g_ = 1.73 for the pCaMKK1 complex and 1.14 at *sR*_g_ = 1.84 for the pCaMKK2 complex, blue and green traces in Figures 4d and S14a,b, respectively) (Kikhney and Svergun, 2015; Receveur‐Brechot and Durand, 2012). Furthermore, the presence of a clear plateau at low angles in the Porod–Debye plot (*s* ^4^ *I*(*s*) vs. *s* ^4^) of the pCaMKK1 complex and its absence from the corresponding plot of the pCaMKK2 complex (Figure S14a,b), as well as the higher Porod–Debye exponent (*P*) (4 for the pCaMKK1 complex and 3.6 for the pCaMKK2 complex) support the hypothesis that the pCaMKK1 complex is less structurally flexible (Rambo and Tainer, 2011).

The dimensionless Kratky plot of 14‐3‐3γ (gray trace in Figure 4d) peaks at 1.104, at a *sR*_g_ value of ∼1.73 consistent with the folded particle (marked by the intersection of the black dotted lines in Figure 4d). In contrast, the profiles of pCaMKK1 and pCaMKK2 alone suggest higher conformational flexibility because they show an outward shift when compared with the profiles of the complexes and 14‐3‐3γ, and the maxima of their curves are higher and at higher *sR*_g_ values (Figures 4d and S14e,g). Moreover, the values of the Porod–Debye exponent also indicate that both pCaMKKs are more structurally flexible than both complexes and 14‐3‐3γ (Figure S14d–g). Our SAXS data analysis showed that unphosphorylated CaMKKs (Table S4 and Figure S13b,d) have higher *R*_g_ and *V*_C_ values than phosphorylated proteins. In contrast, the dimensionless Kratky and Porod–Debye plots indicated a similar degree of structural flexibility (Figure S14d,f). Based on these findings, PKA‐mediated phosphorylation makes both kinases more compact, albeit without affecting their structural flexibility.

Taken together, our model‐independent SAXS data analysis shows that 14‐3‐3γ forms a more compact and less flexible complex with pCaMKK1 than with pCaMKK2, in line with our HDX–MS results.

### 2.6 14‐3‐3γ directly interacts with the N‐lobe of the kinase domain of pCaMKK1

To gain further structural insights into the regulation of CaMKK activity by 14‐3‐3γ, we tried to crystallize the pCaMKK1:14‐3‐3γ complex. 14‐3‐3 protein complexes are difficult to crystalize, mostly due to their conformational heterogeneity, as 14‐3‐3 binding motifs are often found in intrinsically disordered regions of client proteins (Obsilova and Obsil, 2022). Although both HDX–MS and model‐independent SAXS data analysis suggested that pCaMKK1 forms a compact and relatively rigid complex with 14‐3‐3γ, we were unable to obtain crystals of this complex despite extensively screening crystallization conditions. Therefore, SAXS‐based modeling was used to structurally characterize both complexes.

All‐atom modeling of both complexes was performed using the AllosMod‐FoXS method based on molecular dynamics for conformational space sampling (Weinkam et al., 2012). The starting conformation consisted of one CaMKK protomer bound to the 14‐3‐3γ dimer simultaneously through both of its 14‐3‐3 binding motifs. The best‐scoring AllosMod‐FoXS model of the pCaMKK1:14‐3‐3γ complex fitted the experimental scattering data with *χ* ^2^ = 1.18 and placed the kinase domain of pCaMKK1 outside the central channel of the 14‐3‐3γ dimer, close to C‐terminal helices H8 and H9 of one of the 14‐3‐3γ protomers (Figures 4e–g and S15a,b). Overall, the model suggests that 14‐3‐3γ directly interacts with the N‐lobe of the kinase domain of pCaMKK1, more specifically with the β strands that form the binding site for ATP, including the phosphate‐binding loop (P‐loop) located between strands β1 and β2, and the β5‐αE loop involved in the binding of the substrate (Figures 4f and S15b). The binding interface between pCaMKK1 and 14‐3‐3γ mainly consists of conserved residues, especially in 14‐3‐3γ (Figure S16). The shape complementarity of this binding interface was evaluated using the SC program (Lawrence and Colman, 1993). This analysis revealed an average shape complementarity (*S*_C_ = 0.49; interfaces with *S*_C_ = 1 mesh precisely, interfaces with *S*_C_ = 0 are uncorrelated in their topography). However, *S*_C_ was calculated using the crystal structures of individual proteins and, thus, without the induced fit conformational changes that likely accompany complex formation. Therefore, the shape complementarity of the interface between 14‐3‐3γ and pCaMKK1 KD may be higher upon these changes. A more in‐depth analysis of this shape complementarity will require solving the high‐resolution structure of the complex.

The AllosMod‐FoXS model of the pCaMKK1:14‐3‐3γ dimer complex was in line with the results of HDX–MS measurements. After complex formation, significant protection (lower deuteration) was observed in both the N‐lobe of the kinase domain of pCaMKK1 around the ATP‐binding site and 14‐3‐3γ helices H8 and H9 (Figures 2a, 4e,f, and S15a,b). Moreover, this model was consistent with the experimental *R*_g_ and *D*_max_ values (Table S4 and Figure S15c) and with the ab initio shape reconstruction calculated from the scattering data using DAMMIF (Figure 4h). By contrast, the best‐scoring AllosMod‐FoXS model of the pCaMKK2:14‐3‐3γ complex fitted the experimental scattering data with *χ* ^2^ = 2.13 and placed the N‐lobe of the kinase domain of pCaMKK2 at the outer edge of the central channel of the 14‐3‐3γ dimer, with little contacts to this dimer (Figure S17a,b). This model of the pCaMKK2:14‐3‐3γ complex was also consistent with the experimental *R*_g_ and *D*_max_ values (Table S4 and Figure S17c) and with the ab initio shape reconstruction calculated from the scattering data (Figure S17d).

Since the pCaMKK2 complex is more flexible than the pCaMKK1 complex, as indicated by their dimensionless Kratky and Porod–Debye plots (Figures 4d and S14a,b), we also attempted multi‐state modeling of the pCaMKK2:14‐3‐3γ complex using the MultiFoXS method (Schneidman‐Duhovny et al., 2016). However, none of the multi‐state models provided a solution that described the experimental SAXS data better than the single‐state model.

The binding interface between the KD of pCaMKK2 and 14‐3‐3γ was not larger than that of the pCaMKK1 complex, in line with the higher conformational heterogeneity of the complex and with the HDX–MS results. The HDX–MS data had shown significantly weaker deuteration changes in pCaMKK2 than in pCaMKK1 after complex formation (Figures 2b and S17a). These changes in the deuteration of peptides from the C‐terminal helices of 14‐3‐3γ (especially at longer deuteration times) and in several regions of the kinase domain of pCaMKK2 likely result from transient contacts between 14‐3‐3γ and the kinase domain and/or flexible segments of pCaMKK2 located on both sides of its kinase domain.

The theoretical scattering curve calculated from the crystal structure of 14‐3‐3γ fit the experimental data well (*χ* ^2^ = 1.35, Figure S18c). In addition, the superposition of the calculated ab initio molecular envelope with the crystal structure of 14‐3‐3γ correctly reproduced its molecular shape (Figure S18a,b,d). SAXS data analysis of CaMKKs alone suggested that both kinases in the unphosphorylated and phosphorylated states are more structurally flexible than both complexes and 14‐3‐3γ (Figure S14). For this reason, ab initio shape reconstruction was not performed for these proteins because only one conformation is used to describe a flexible molecule (Bernado and Svergun, 2012).

To gain further insights into the structural behavior of CaMKKs, all‐atom modeling of CaMKK2 was performed using AllosMod‐FoXS and MultiFoXS. CaMKK2 was selected based on the better quality (lower noise) of its scattering data, which were collected at a higher protein concentration (Table S4). The resulting best‐scoring model was based on a weighted combination of three states and fit the experimental SAXS data with a *χ* ^2^ of 1.75 (Figure S19). Models based on one or a combination of two states provided a much poorer fit to the SAXS data than the three‐state model. Accordingly, CaMKKs should be highly conformationally heterogeneous in solution, with their N‐ and C‐terminal segments sampling different conformations, as indicated by dimensionless Kratky and Porod–Debye plots (Figure S14d–g).

## 3 DISCUSSION

Phosphorylation by PKA partly inhibits the activity of both CaMKKs, thus enabling the recruitment of adaptor proteins 14‐3‐3. 14‐3‐3 proteins further modulate the kinase activity of both enzymes through different mechanisms. pCaMKK1 is fully inhibited by 14‐3‐3 binding, whereas pCaMKK2 activity remains almost unchanged (Figure 1h,i). To clarify this difference, we performed a detailed biophysical and structural analysis of both complexes.

14‐3‐3 binding inhibits the catalytic activity of pCaMKK1 by decreasing its *V*_max_ (Ichimura et al., 2008). Our SAXS‐based structural analysis suggested that the active site and the segment involved in the substrate binding of the kinase domain of pCaMKK1 directly interacts with the C‐terminal portion of 14‐3‐3γ (Figures 4e,f and S15). The involvement of the last two C‐terminal helices of the 14‐3‐3γ molecule in pCaMKK1 binding is not surprising since these two highly conserved helices (Figure S16) are often involved in interactions with binding partners, as demonstrated by previously solved structures of 14‐3‐3 protein complexes with AANAT (Obsil et al., 2001), Nth1 (Alblova et al., 2017), B‐RAF:MEK1 (Park et al., 2019), and ExoS (Karlberg et al., 2018) (Figure S20). For example, in complexes with yeast neutral trehalase Nth1 (Figure S20b) and Pseudomonas exotoxin‐S (ExoS) (Figure S20d), the last two C‐terminal helices H8 and H9 of the 14‐3‐3 protein form most of the binding interface. Thus, we hypothesized that this interaction affects the structure of the active center of the kinase domain and blocks substrate binding.

This hypothesis was supported by our HDX–MS data, which revealed a considerably lower deuteration in the N‐lobe of the kinase domain and especially in β strands forming the ATP‐binding pocket including the phosphate‐binding P‐loop (Figures 2a, 4f, and S15b). Furthermore, 14‐3‐3γ binding effectively blocked the interaction between pCaMKK1 and Ca^2+^/CaM, which is only partly inhibited by phosphorylation alone (Figure 3a,e). Therefore, Ca^2+^/CaM displacement from pCaMKK1 is likely caused by steric hindrance due to the close proximity of the CBD region to the C‐terminal 14‐3‐3‐binding motif and thus to the 14‐3‐3γ surface (Figures 4e and S15).

In line with the above, deuterium uptake significantly decreased throughout the autoregulatory segment of pCaMKK1, including CBD, upon 14‐3‐3γ binding (Figures 2a, 4e, S8, and S15). The direct inhibitory effect of 14‐3‐3 binding on pCaMKK1 activity may be explained by structural modulation and obstruction of the active site and by blocking of Ca^2+^/CaM binding to the autoregulatory segment. 14‐3‐3 binding may also protect the inhibitory phosphorylation site of Ser^458^ in CBD from dephosphorylation, as previously shown by Davare et al. (2004) and supported by the lower deuteration in this region (Figures 2a and S8). This protection against dephosphorylation should help to maintain pCaMKK1 in its inhibited state mediated by PKA.

14‐3‐3 binding does not appear to have a significant direct effect on the catalytic activity of pCaMKK2 (Figure 1i) (Psenakova et al., 2018). This result is consistent with the absence of a large binding interface between pCaMKK2 and 14‐3‐3γ, as suggested by our SAXS‐based model, and with deuteration changes, which were significantly weaker in pCaMKK2 than in pCaMKK1, after complex formation (Figures 2b and S17). Both our previous study on the interaction of pCaMKK2 with 14‐3‐3γ (Psenakova et al., 2018) and a study recently published by Langendorf et al. (2020) reported that phosphorylation‐mediated 14‐3‐3 protein recruitment keeps pCaMKK2 in a partly inhibited state by protecting the inhibitory phosphorylation site Ser^495^ in CBD from dephosphorylation. Our HDX–MS analysis revealed lower deuterium uptake in this region of pCaMKK2, thus corroborating these studies (Figure 2b).

The fast deuteration of the N‐ and C‐terminal segment of CaMKKs (black traces in Figures S8 and S10), together with SAXS data analysis and CaMKK2 modeling (Figures S14 and S19), indicated that these segments are well exposed to the solvent and highly flexible. Except for the AID/CBD region, 14‐3‐3γ binding had little effect on the deuteration of these segments (Figure 2a,b), so they should remain solvent accessible and/or flexible after complex formation. In fact, significant portions of these segments remain exposed to the solvent according to our AllosMod‐FoXS models of both complexes (Figures 4e, S15, and S17). The phosphorylation site located in CBD (Ser^458^ in CaMKK1 and Ser^495^ in CaMKK2) is protected against dephosphorylation most likely due to the proximity of the 14‐3‐3γ surface, which sterically hinders access to this region.

The exact reason for these differences in 14‐3‐3 interactions with pCaMKK1 and 2 is still unclear. A possible explanation is the difference in the length of the unstructured segments between the N‐terminal 14‐3‐3 binding motif and the kinase domain (Figure S1). This segment is 11 amino acids longer in CaMKK2 than in CaMKK1 (in CaMKK1_67–480_ D293A and CaMKK2_93–517_ D330A used in this study), which could affect the position of the kinase domain of pCaMKK2 in a complex with 14‐3‐3γ. The shorter segment of pCaMKK1 may reduce the conformational flexibility and enable the N‐lobe of the kinase domain to interact with the C‐terminal helices of the 14‐3‐3γ protein. These conformational differences between pCaMKK1 and pCaMKK2 in a complex with 14‐3‐3γ, more specifically the lower *R*_g_ and *V*_C_ values and the higher Porod–Debye exponent of the pCaMKK1 complex, are also indicated by the SAXS data analysis (Table S4 and Figures 4d and S14a,b) and by the HDX–MS measurements (Figure 2a,b).

Another likely factor may be differences in the sequence of unstructured segments, particularly the N‐terminal segment that separates the N‐terminal 14‐3‐3‐binding motif and the kinase domain (Figure S1). Furthermore, our previous analysis of the complex between pCaMKK2 with only the N‐terminal 14‐3‐3 binding motif (Ser^100^) and 14‐3‐3γ also positioned the kinase domain outside the central channel of the 14‐3‐3γ dimer, but in a different orientation, with the N‐lobe of the kinase domain close to the loop between 14‐3‐3γ helices H4 and H5 (Psenakova et al., 2018). Based on this evidence, pCaMKKs with different levels of phosphorylation and hence different numbers of functional 14‐3‐3 binding motifs may interact with the 14‐3‐3 dimer in markedly different ways, which in turn differently affect their activity.

In conclusion, both phosphorylated CaMKKs interact with the 14‐3‐3γ protein at similarly high binding affinities and form complexes in which one pCaMKK molecule binds to the 14‐3‐3γ dimer. Complex formation suppresses the interaction of both pCaMKKs with Ca^2+^/CaM and affects the structure and/or the accessibility of their kinase domains and autoinhibitory segments. However, these effects are much stronger on pCaMKK1 than on pCaMKK2 because the pCaMKK1:14‐3‐3γ complex is more compact and rigid. In this complex, the active site of pCaMKK1 interacts with the last two C‐terminal helices of the 14‐3‐3γ protein. Combined with the suppression of Ca^2+^/CaM binding, this interaction provides a structural basis for 14‐3‐3‐dependent pCaMKK1 inhibition. Conversely, the loosened and more flexible structure of the pCaMKK2:14‐3‐3 complex explains the weak effect of 14‐3‐3 binding on the catalytic activity of pCaMKK2.

Further studies are needed to assess whether the interaction with 14‐3‐3 can be used as an alternative approach to modulating CaMKKs‐mediated signaling. Since a recent report on the stabilization of interactions between pCaMKK2 and 14‐3‐3 by fusicoccins has suggested that this approach is feasible (Lentini Santo et al., 2020), our findings may help to further exploit these key protein–protein interactions in the targeted regulation of both CaMKKs.

## 4 MATERIALS AND METHODS

### 4.1 Expression, purification and phosphorylation of human CaMKK1 and CaMKK2

DNA encoding human CaMKK1 (residues 67–480) was ligated into pRSFDuet‐1 (Novagen) using NcoI and NotI sites. Expression constructs of CaMKK2 (residues 93–517) and its mutant variants were described previously (Psenakova et al., 2018). All CaMKK1 mutants were generated using the QuikChange site‐directed mutagenesis kit (Stratagene, La Jolla, CA, USA), and the mutations were confirmed by sequencing. Both CaMKK1 and CaMKK2 were expressed as N‐terminal 6× His‐GB1‐tagged fusion proteins in *Escherichia coli* BL21 (DE3) cells, as described previously (Psenakova et al., 2018). Purified CaMKK1 and CaMKK2 were phosphorylated in the presence of 20 mM MgCl_2_ and 0.75 mM ATP by incubation with 2000 U (2700 U in the case of CaMKK2 T146A, D330A, and S945A variant) of PKA per mg of protein. After phosphorylation, ATP and PKA were removed by size‐exclusion chromatography on a HiLoad Superdex 200 pg 26/600 (GE Healthcare, Chicago, IL, USA) in buffer containing 20 mM Tris–HCl (pH 7.5), 150 mM NaCl, 5 mM DTT, and 10% (w/v) glycerol. The results of the phosphorylation reaction were analyzed by HPLC–MS.

### 4.2 Expression and purification of 14‐3‐3 proteins

Human 14‐3‐3γ (residues 1–235 lacking the C‐terminal 12‐residues‐long flexible tail) was expressed and purified as described previously (Obsilova et al., 2004).

### 4.3 Expression and purification of CaM

Rat CaM was prepared as described previously (Holakovska et al., 2011).

### 4.4 Preparation of kinase‐dead CaMK1D

The kinase‐dead CaMK1D D^165^A was expressed and purified as described previously (Psenakova et al., 2018).

### 4.5 CaM dansyl labeling

CaM was labeled by dansyl chloride as described previously (Kincaid et al., 1982). Briefly, the required amount of CaM was dialyzed against 10 mM NaHCO_3_ (pH 10.0) and diluted to 1 mg mL^−1^. After drop‐wise addition of dansyl chloride (Sigma‐Aldrich, St. Louis, MO, USA) from a 6 mM acetone stock to a final concentration of 90 μM, the sample was incubated for 45 min at 30°C and then for another 18 h at 8°C. The excess of dansyl chloride was removed by size exclusion chromatography on a HiLoad Superdex 75 PG 26/600 column (GE Healthcare, Chicago, IL, USA) in a buffer containing 20 mM HEPES–NaOH (pH 7.5), 150 mM NaCl, 5 mM DTT, and 10% (w/v) glycerol at pH 7.5. The efficiency of the reaction was evaluated as the ratio between the concentration of CaM and dansyl calculated from the absorbance at 280 and 333 nm, respectively.

### 4.6 Enzyme activity measurements

The kinase activity of CaMKK1 and CaMKK2 were examined using a γ‐[^32^P]‐ATP assay with inactive CaMK1D D165A as a physiological kinase substrate. The reaction volume of 45 μL consisted of 1 nM CaMKK1 (or 2 nM CaMKK2_93–517_ respectively), 30 μM CaMK1D D165A, 5 μM CaM, and 10 μM 14‐3‐3γ (where needed) in buffer containing 50 mM HEPES (pH 7.5), 200 mM NaCl, 20 mM MgCl_2_, 1 mM CaCl_2_, 1 mM DTT, and 10% glycerol. The reaction was heated to 30°C and initiated by adding 5 μL of 1 mM γ‐[^32^P]‐ATP (PerkinElmer Life Sciences, Waltham, MA, USA) to a final ATP concentration 100 μM (~2–5 μCi per reaction). After a 5‐min incubation at 30°C, the reaction was stopped by spotting 45 μL of the reaction mixture onto a P81 phosphocellulose paper strip (Merck Millipore, Darmstadt, Germany) and washing in 3× 500 mL of 1% (w/v) phosphoric acid for 5 min each. Strips were dried and inserted into vials containing 5 mL of scintillation fluid (Rotiszint™, Carl Roth BmbH, Karlsruhe, Germany). Counts were performed on a Quantasmart™ liquid analyzer (PerkinElmer Life Sciences, Waltham, MA, USA). The curve of product formation as a function of time confirmed that the data were collected under linear assay conditions, with conversion rates of both ATP and the second substrate CaMK1D D165A lower than 5%. The significance of the changes was assessed according to a paired two‐tail *t* test, where indicated in the figures, * denotes *p* ≤ 0.05, and ** denotes *p* ≤ 0.005.

### 4.7 HPLC–MS analysis of CaMKKs

HPLC–MS analysis was performed as described previously (Horvath et al., 2021).

### 4.8 Analytical ultracentrifugation

Sedimentation velocity (SV) experiments were performed on a ProteomLabTM XL‐I analytical ultracentrifuge (Beckman Coulter, Brea, CA, USA), as previously described (Petrvalska et al., 2016). Samples were dialyzed against a buffer containing 50 mM Tris–HCl (pH 7.5), 150 mM NaCl, 1 mM tris(2‐carboxyethyl)phosphine (TCEP), and 1 mM CaCl_2_ (where needed) before analysis. SV experiments were conducted in charcoal‐filled Epon centerpieces with a 12‐mm optical path length, at 20°C, and at 42,000 rpm rotor speed (An‐50 Ti rotor, Beckman Coulter, Brea, CA, USA). All sedimentation profiles were recorded with absorption optics. Mixtures of pCaMKK1 or pCaMKK2 with 14‐3‐3γ were analyzed at various molar ratios, with 0.06–6 μM pCaMKK1 or pCaMKK2 and 1.2 μM 14‐3‐3γ. SV AUC data were analyzed using the programs Sedfit and Sedphat (Schuck, 2000; Houtman et al., 2007).

### 4.9 Small angle X‐ray scattering

Synchrotron SAXS data were collected at beamline P12 operated by EMBL Hamburg at the PETRA III storage ring (DESY, Hamburg, Germany). All proteins were dialyzed in a buffer containing 50 mM Tris–HCl (pH 7.5), 150 mM NaCl, 1 mM TCEP, and 3% (w/v) glycerol. The scattering data of 14‐3‐3γ, CaMKK1, pCaMKK1, CaMKK2, pCaMKK2, and the pCaMKK1:14‐3‐3γ and pCaMKK2:14‐3‐3γ complexes (mixed in a 1:2 molar ratio) were collected in in‐line SEC‐SAXS mode on a Superdex 200 Increase 5/150 GL column (GE Healthcare, Chicago, IL, USA) at a flow rate of 0.3 mL min^−1^. The forward scattering *I*_0_ and the radius of gyration *R*_*g*_ were calculated using the Guinier approximation for the *s* (*s* = 4*π*sin(*θ*)/*λ*, where 2*θ* is the scattering angle, and *λ* is the wavelength) range that satisfies the *sR*_*g*_ < 1.3 condition. SEC‐SAXS data were processed using CHROMIXS (Panjkovich and Svergun, 2018). Data were selected based on the constant *R*_g_ where possible. The distance distribution functions *P*(*r*) and the maximum particle dimensions *D*_max_ were calculated using the program GNOM (Svergun, 1992). The excluded volume of the hydrated particle (the Porod volume, *V*_P_) was calculated using the program PRIMUS (Konarev et al., 2003). The Porod–Debye analysis and *V*_C_ calculation were performed using the program ScÅtter IV (https://bl1231.als.lbl.gov/scatter/). The program DAMMIF (Franke and Svergun, 2009) was used to calculate ab initio molecular envelopes. Fifteen iterations of DAMMIF were averaged using the program DAMAVER (Volkov and Svergun, 2003), and the averaged and filtered envelope was refined using one run of DAMMIN (Svergun, 1999). The calculated molecular envelope was aligned to structural models using SUPCOMB (Kozin and Svergun, 2001). Theoretical scattering curves were calculated from structural models and fitted to experimental scattering data using CRYSOL (Svergun et al., 1995) and FoXS (Schneidman‐Duhovny et al., 2013).

### 4.10 All‐atom modeling of CaMKK1/2 and the pCaMKK1/2:14‐3‐3γ complexes using SAXS data

All‐atom modeling of the pCaMKK1/2:14‐3‐3γ complexes was performed using the AllosMod‐FoXS method (Weinkam et al., 2012; Schneidman‐Duhovny et al., 2010). The starting model was generated using the crystal structures of 14‐3‐3γ with bound CaMKK phosphopeptides (PDB ID: 6FEL and 6EWW (Psenakova et al., 2018)) and catalytic domains of CaMKK1 (PDB ID: 6CD6) and CaMKK2 (PDB ID: 5UY6). The N‐ and C‐terminal 14‐3‐3 binding motif of pCaMKKs were restrained in the ligand binding groove of 14‐3‐3γ, in the orientation observed in structures of the 14‐3‐3γ:phosphopeptide complexes (Psenakova et al., 2018). AllosMod simulations consisting of 10–30 runs (generating 101 conformations for each run) sampled the most probable conformations consistent with the starting structures. The *χ* ^2^ values were used to select the most consistent models with the SAXS data. Multistate modeling of the pCaMKK2:14‐3‐3γ complex and CaMKK2 were performed using the MultiFoXS (Schneidman‐Duhovny et al., 2016).

### 4.11 Fluorescence polarization assay

Fluorescence polarization measurements were performed on a CLARIOstar microplate reader (BMG Labtech, Germany) using 384‐well, black, low‐volume, flat‐bottom plates (Corning, USA) with 2 μM DANS‐CaM in a buffer containing 10 mM HEPES–NaOH (pH 7.4), 150 mM NaCl, 1 mM CaCl_2_, 0.1% (v/v) Tween 20, and 0.1% (w/v) BSA. The excitation and emission wavelengths were 340 and 530 nm, respectively. In addition, 30 μM CaMKK1 or CaMKK2 proteins and their binary dilution series were incubated for 1 h with 2 μM DANS‐CaM and 60 μM 14‐3‐3γ (where needed), before the fluorescence polarization measurements. To determine the *K*_D_ values, the resulting curves were fitted to a one‐site‐binding model using GraphPad Prism 9.5.1 (GraphPad Software, La Jolla, CA, USA).

### 4.12 Time‐resolved fluorescence measurements

Time‐resolved dansyl fluorescence intensity and anisotropy decay measurements, as well as data analysis, were performed as described previously (Horvath et al., 2021). Dansyl emission was excited at 355 nm by the doubled and pulse‐picked output of the Ti:sapphire laser, and the emission was isolated at 540 nm using a combination of monochromator and a dielectric long‐pass filter with a cut‐off wavelength of 520 nm (Chroma, USA) placed in front of its input slit. The experimental decays were deconvolved using the model‐independent maximum entropy method (MEM) (Vecer and Herman, 2011; Bryan, 1990). Samples were placed in a thermostatic holder, and all experiments were performed at 23°C in a buffer containing 50 mM Tris–HCl (pH 7.5), 150 mM NaCl, 1 mM TCEP, 1 mM CaCl_2_, and 10% (w/v) glycerol. The DANS‐CaM, CaMKK1, CaMKK2, and 14‐3‐3γ concentrations were 20, 23, 23, and 100 μM, respectively.

### 4.13 Hydrogen–deuterium exchange coupled to MS

HDX kinetics was followed separately on two protein complexes, pCaMKK1:14‐3‐3γ and pCaMKK2:14‐3‐3γ, after 20 s, 2 min, 20 min, and 2 h of incubation in a D_2_O‐based buffer containing 50 mM Tris (pH 7.5), 0.5 M NaCl, 5 mM DTT, and 10% glycerol. For the pCaMKK2:14‐3‐3γ complex, all measurements were performed in triplicates, at all time points. For CaMKK1‐1433 HDX, only the measurements at 20 s and 2 h time were performed in triplicates. HDX was initiated by diluting the 80 μM protein sample 10× in D_2_O‐based buffer. After incubation, HDX was quenched by adding 0.5 M glycine–HCl, pH 2.3 at a 1:1 ratio. Immediately, the sample was frozen in liquid nitrogen. Subsequently, each sample was thawed and injected into the LC system, including an immobilized nepenthesin‐2 protease column, where the sample was delivered in a flow of 0.4% formic acid (FA) in water, at 400 μL min^−1^ flow rate (1260 Infinity II Quaternary pump, Agilent Technologies, Waldbronn, Germany). The peptides were online trapped and desalted on a SecurityGuard pre‐column (ULTRA Cartridges UHPLC Fully Porous Polar C18, 2.1 mm, Phenomenex, Torrance, CA, USA) and separated on a reversed‐phase analytical column (LUNA Omega Polar C18 Column, 100 Å, 1.6 μm, 100 mm × 1.0 mm, Phenomenex, Torrance, CA, USA) at a flow rate of 40 μL min^−1^ using a 10–40% linear gradient of solvent B (A: 2% acetonitrile/0.1% FA in water; B: 98% acetonitrile/0.1% FA in water) (1290 Infinity II LC system, Agilent Technologies, Waldbronn, Germany). Digestion and separation steps were performed at 0°C and pH 2.3 to minimize deuterium loss. The LC system was directly interfaced with an ESI source of 15 T FT‐ICR mass spectrometer (SolariX XR, Bruker Daltonics, Bremen, Germany). Data were exported and processed using Data Analysis version 5.3 (Bruker Daltonics, Bremen, Germany) and in‐house developed DeutEx software (https://deutex.org). The peptides were identified for each variant by data‐dependent LC–MS/MS analysis using the same LC system and gradient elution in a MASCOT (Matrix Science, London, UK) search against a custom‐built database combining sequences of our proteins and contaminants from the cRAP database.

## AUTHOR CONTRIBUTIONS

Tomas Obsil and Veronika Obsilova supervised the project and provided scientific guidance. Olivia Petrvalska performed protein expression/purification experiments, analytical ultracentrifugation experiments, kinase activity measurements, FP measurements and prepared samples for SAXS, HDX–MS and fluorescence experiments. Nicola Koupilova performed protein expression/purification experiments and optimized the phosphorylation protocol of CaMKKs. Karolina Honzejkova performed protein expression/purification experiments and processed and analyzed SAXS data. Petr Herman performed time‐resolved fluorescence measurements and data analysis, and Veronika Obsilova and Tomas Obsil wrote the manuscript. All co‐authors revised the manuscript.

## FUNDING INFORMATION

This study was supported by the Czech Science Foundation (Tomas Obsil, grant number 19‐00121S, Petr Herman, grant number 22‐03875S) and the Grant Agency of the Charles University (Karolina Honzejkova, grant number 1160120). We thank the Czech Infrastructure for Integrative Structural Biology (CIISB) for access to CMS facilities at BIOCEV (project LM2018127 by MEYS).

## CONFLICT OF INTEREST STATEMENT

The authors declare no conflicts of interest.

## Supporting information


**Appendix S1:** Supporting Information
