# Supplementary material for: 14‐3‐3 protein inhibits CaMKK1 by blocking the kinase active site with its last two C‐terminal helices
Source: Protein Sci. 2023 Nov 1;32(11):e4805. doi: 10.1002/pro.4805 (PMC10588359; doi:10.1002/pro.4805)
Supplement: Supplementary file 1 — Appendix S1: Supporting Information [file PRO-32-e4805-s001.pdf]

Supplementary Material

14-3-3 protein inhibits CaMKK1 by blocking the kinase active site with its last two C-terminal helices

Olivia Petrvalska[1,2,#], Karolina Honzejkova[1,#], Nicola Koupilova[1], Petr Herman[3], Veronika Obsilova[2], and Tomas Obsil[1,2]

[1] Department of Physical and Macromolecular Chemistry, Faculty of Science, Charles University, Prague, Czech Republic
[2] Institute of Physiology of the Czech Academy of Sciences, Laboratory of Structural Biology of Signaling Proteins, Division BIOCEV, Vestec, Czech Republic
[3] Institute of Physics, Faculty of Mathematics and Physics, Charles University, Prague, Czech Republic

[#] Contributed equally

Correspondence: Tomas Obsil, Faculty of Science, Charles University, Albertov 6, Prague, 12843, Czech Republic, Email: obsil@natur.cuni.cz, Tel. +420-221951303. Veronika Obsilova, Institute of Physiology, CAS, Division BIOCEV, Prumyslova 595, Vestec, 25250 Czech Republic, Email: veronika.obsilova@fgu.cas.cz, Tel.: +420-325873513

Supplemental Table S1: Sedimentation coefficient values and estimated molecule weights from SV AUC measurements.

Sample	*s* (S)[a]	M_w (kDa)	f/f_0	Theoretical M_w (kDa)
CaMKK1 (protomer)[b]	2.88 ± 0.01	~39	1.32	46.6
CaMKK1 (dimer)[b]	4.14 ± 0.04	~58	1.32	93.2
**p**CaMKK1 (protomer)[b]	3.38 ± 0.03	~40	1.28	46.9
CaMKK2 (protomer)[b]	3.20 ± 0.03	~42	1.35	47.6
**p**CaMKK2 (protomer)[b]	3.21 ± 0.03	~44	1.4	47.9
CaMKK1:Ca^{2+}/CaM (1:1)[b]	3.80 ± 0.03	~58	1.43	63.4
CaMKK2:Ca^{2+}/CaM (1:1)[b]	3.90 ± 0.04	~57	1.36	64.4
**p**CaMKK1:14-3-3γ (1:2)[c]	5.57 ± 0.05	~93	1.33	101.2
**p**CaMKK2:14-3-3γ (1:2)[c]	5.78 ± 0.02	~102	1.36	102.2

[a]Analysis of SV AUC data was performed using the programs Sedfit and Sedphat (1; 2).
[b]For samples with a concentration of 10 μM.
[c]For data obtained at the highest concentration of pCaMKKs (1.2 μM 14-3-3γ and 6 μM pCaMKKs).

Supplemental Table S2: Binding affinities of Dans-CaM for CaMKKs assessed by fluorescence polarization

Sample	K_D (μM)
**4×Phosphorylated CaMKKs**	
CaMKK1	0.60 ± 0.11
CaMKK1 + 14-3-3γ	0.63 ± 0.07
**p**CaMKK1	2.16 ± 0.30
**p**CaMKK1 + 14-3-3γ	-
CaMKK2	0.56 ± 0.09
CaMKK2 + 14-3-3γ	0.51 ± 0.10
**p**CaMKK2	3.55 ± 0.93
**p**CaMKK2 + 14-3-3γ	> 10.75
**2×Phosphorylated CaMKKs (only at 14-3-3 binding motifs)**	
CaMKK1	0.53 ± 0.05
CaMKK1 + 14-3-3γ	0.42 ± 0.04
**p**CaMKK1	0.53 ± 0.04
**p**CaMKK1 + 14-3-3γ	3.18 ± 0.49
CaMKK2	0.56 ± 0.07
CaMKK2 + 14-3-3γ	0.52 ± 0.06
**p**CaMKK2	0.53 ± 0.04
**p**CaMKK2 + 14-3-3γ	2.77 ± 0.30

Supplemental Table S3: Summary of time-resolved Dans-CaM fluorescence measurements

Sample	τ_{mean}[a,b] (ns)	ϕ_1 (ns)	β_1[c]	ϕ_2 (ns)	β_2	ϕ_3 (ns)	β_3	ϕ_4 (ns)	β_4
CaM	15.7	1.8	0.09	7.2	0.16	25	0.03		
CaM+CaMKK1	19.9	2.1	0.03	5.6	0.02	19	0.13	80	0.15
CaM+CaMKK1+14-3-3γ	19.9	2.8	0.04	13.7	0.08	52	0.19	>200	0.01
CaM+**p**CaMKK1	20.2	2.1	0.04	9.3	0.10	63	0.18		
CaM+**p**CaMKK1+14-3-3γ	17.5	1.8	0.07	7.0	0.15	27	0.04	184	0.03
CaM+CaMKK2	20.1	1.5	0.02	4.2	0.03	17	0.11	57	0.15
CaM+CaMKK2+14-3-3γ	20.1	2.5	0.03	10.7	0.06	38	0.19	185	0.02
CaM+**p**CaMKK2	20.0	2.8	0.06	13.0	0.10	61	0.15		
CaM+**p**CaMKK2+14-3-3γ	18.2	2.2	0.07	7.2	0.10	23	0.04	184	0.08

[a]Mean lifetimes were calculated from the fitted lifetime distribution as $\tau_{mean}=\Sigma_i f_i \tau_i$, where f_i is an intensity fraction of the *i*-th lifetime component τ_i (3; 4).

[b]SD = ± 0.1 ns

[c]The emission anisotropies $r(t)$ were analyzed for a series of exponentials using a model-independent maximum entropy method without any assumptions about the shape of the anisotropy decay and the correlation time distribution (3; 4), $r(t) = \Sigma_i \beta_i' \exp(-t/\phi_i')$, where the amplitudes β_i' represent the distribution of the correlation times ϕ_i'. The β_i values in the table represent peaks of the distribution positioned at the correlation times ϕ_i.

Supplemental Table S4: SAS data acquisition, sample details, data analysis, modelling, fitting and software used for SAS data reduction, analysis and interpretation

**Sample details**							
	14-3-3γΔC (dimer)	CaMKK1	pCaMKK1	CaMKK2	pCaMKK2	pCaMKK1: 14-3-3γΔC (1:2)	pCaMKK2: 14-3-3γΔC (1:2)
Organism	Human	Human	Human	Human	Human	Human	Human
Source (Catalogue No. or reference)	*E. coli*	*E. coli*	*E. coli*	*E. coli*	*E. coli*	*E. coli*	*E. coli*
Uniprot ID	P61981	Q8N5S9	Q8N5S9	Q96RR4	Q96RR4	Q8N5S9, P61981	Q96RR4, P61981
Residues	1-235	67-480	67-480	93-517	93-517	67-480, 1-235	93-517, 1-235
Extinction coefficient ε (M^{-1} cm^{-1})	31 860	41 370	41 370	28 880	28 880	105 090	92 600
Molecular mass *M* from chemical composition (kDa)	54.3	46.6	46.9	47.6	47.9	101.2	102.2
Loading concentration (mg mL^{-1})	6.0	3.2	3.0	9.4	5.5	25.5	21.5
Injection volume (µl)	48	50	50	50	50	50	50
Flow rate (ml min^{-1})	0.3	0.3	0.3	0.3	0.3	0.3	0.3
Solvent composition and source	50 mM Tris-HCl (pH 7.5), 150 mM NaCl, 1 mM TCEP and 3% (w/v) glycerol						
**SAS data collection parameters**							
Instrument	DESY, the P12 beamline. Source: Petra III U29 undulator, Monochromator: Double crystal Si (111), Detector: Pilatus 6M (https://www.embl-hamburg.de/biosaxs/p12/characteristics.html).						
Wavelength (Å)	1.2398						
Sample-to-detector distance (m)	3						
Beam size (mm^2)	0.2 × 0.05 at the detector						
s-measurement range ($Å^{-1}$)	0.0025-0.732	0.0023-0.443	0.0023-0.443	0.0025-0.732	0.0023-0.443	0.0024-0.737	0.0024-0.737
Absolute scaling method	Water						
Basis for normalization to constant counts	To transmitted intensity measured at the beam-stop.						
Sample configuration	SEC-SAXS. Size separation used a Superdex 200 Increase 5/150 GL column.						
Exposure time (s)	0.495	0.495	0.495	0.495	0.495	0.495	0.495
Exposure period (s)	0.5	0.5	0.5	0.5	0.5	0.5	0.5
Sample temperature (ºC)	20	20	20	20	20	20	20

Software used for SAS data reduction, analysis and interpretation							
SAS data reduction	SASFLOW (5)						
Basic analyses: Guinier, $P(r)$, Porod volume V_P, M.W., Porod exponent (P)	PRIMUS/qt ATSAS v2.8.4 and v3.2.1 (6)<br>ScÅtter IV (https://bl1231.als.lbl.gov/scatter/), SAXSMoW (7)						
ε from sequence	ExPASy - ProtParam Tool						
Shape/bead modelling	DAMMIF and DAMMIN ATSAS v3.2.1 (8)						
Atomic structure modelling (homology, rigid body, ensemble)	CORAL ATSAS v.3.2.1 (9), AllosMod-FoXS (10), MultiFoXS (11), HYDROPRO 10 (12)						
Modelling of missing sequence from PDB files	COOT v.0.9.8.7 (13)						
Molecular graphics	PyMOL (https://pymol.org/2/)						
**Structural parameters**							
Guinier Analysis							
$I(0)$ (cm^{-1})	0.06	0.015	0.02	0.064	0.041	0.283	0.253
R_g (Å)	28.4	31.5	29.5	31.7	30.1	33.5	36.5
s-range (Å^{-1})	0.0081-0.0456	0.0164-0.0412	0.017-0.0441	0.01-0.0331	0.0144-0.0406	0.0069-0.0388	0.0044-0.0354
P(r) analysis							
$I(0)$ (cm^{-1})	0.060	0.015	0.02	0.064	0.041	0.283	0.254
R_g (Å)	28.4	32.2	30.2	32.9	31.4	33.3	37
D_{max} (Å)	84	107	105	133	120	108	132
s-range (Å^{-1})	0.0081-0.2812	0.0164-0.254	0.017-0.272	0.01-0.252	0.0144-0.266	0.0069-0.2389	0.0044-0.2192
Other parameters							
Porod volume V_P (Å^3)	76600	96500	87600	91000	88600	152000	156800
Volume-of-correlation V_C (Å^2)	423	438	424	453	434	637	662
M from a consensus Bayesian assessment method (kDa)	50.9	53.2	47.7	55.6	53.2	101.1	94.2
**Shape modelling results**							
s-range for fitting (Å^{-1})	0.0081-0.2815					0.006-0.2386	0.0044-0.219
Symmetry/anisotropy assumptions	P2					P1	P1
χ^2 value	1.063					1.158	1.115

**Atomistic modelling**							
Method				MultiFoXS		AllosMod-FoxS	AllosMod-FoxS
s-range for fitting (Å^{-1})				0.0025-0.5		0.003-0.5	0.0024-0.5
Symmetry assumptions				N/A		N/A	N/A
χ^2 value				1.75		1.18	2.13
Weights for multi-state models				Best scoring 3-state model: w_1 = 29% w_2 = 45% w_3 = 26%		N/A	N/A
Predicted R_g (Å)				Model 1: 37.1 Model 2: 28.8 Model 3: 34.4		33.2	37.0
Predicted D_{max} (Å)				Model 1: 145 Model 2: 103 Model 3: 143		116	130
**Data and model deposition IDs**	SASDSJ7	SASDSR8	SASDSS8	SASDSQ7	SASDST8	SASDS46	SASDSX7
**Frames Averaged**	725-774	610-669	766-797	722-748	783-834	622-659	726-759
**Buffer Frames Used**	350-450	931-999	957-1011	304-336	1028-1109	356-444	544-612

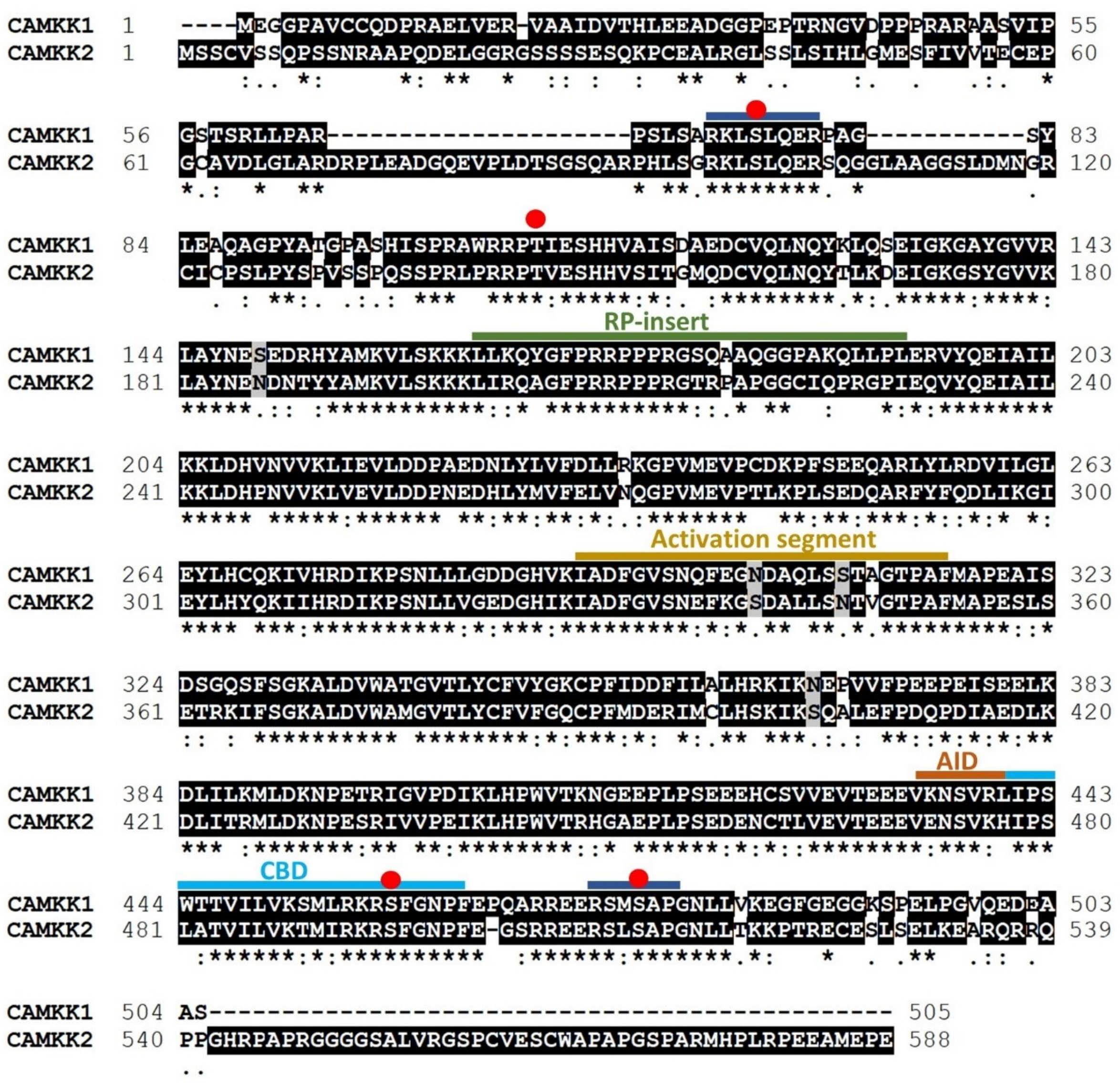

Supplemental Figure S1. Sequence alignment of human CaMKK1 (Q8N5S9) and CaMKK2 (Q96RR4) using the CLUSTALW server (https://www.genome.jp/tools-bin/clustalw); 14-3-3 binding motifs are indicated by dark blue lines, and PKA phosphorylation sites are indicated by red circles.

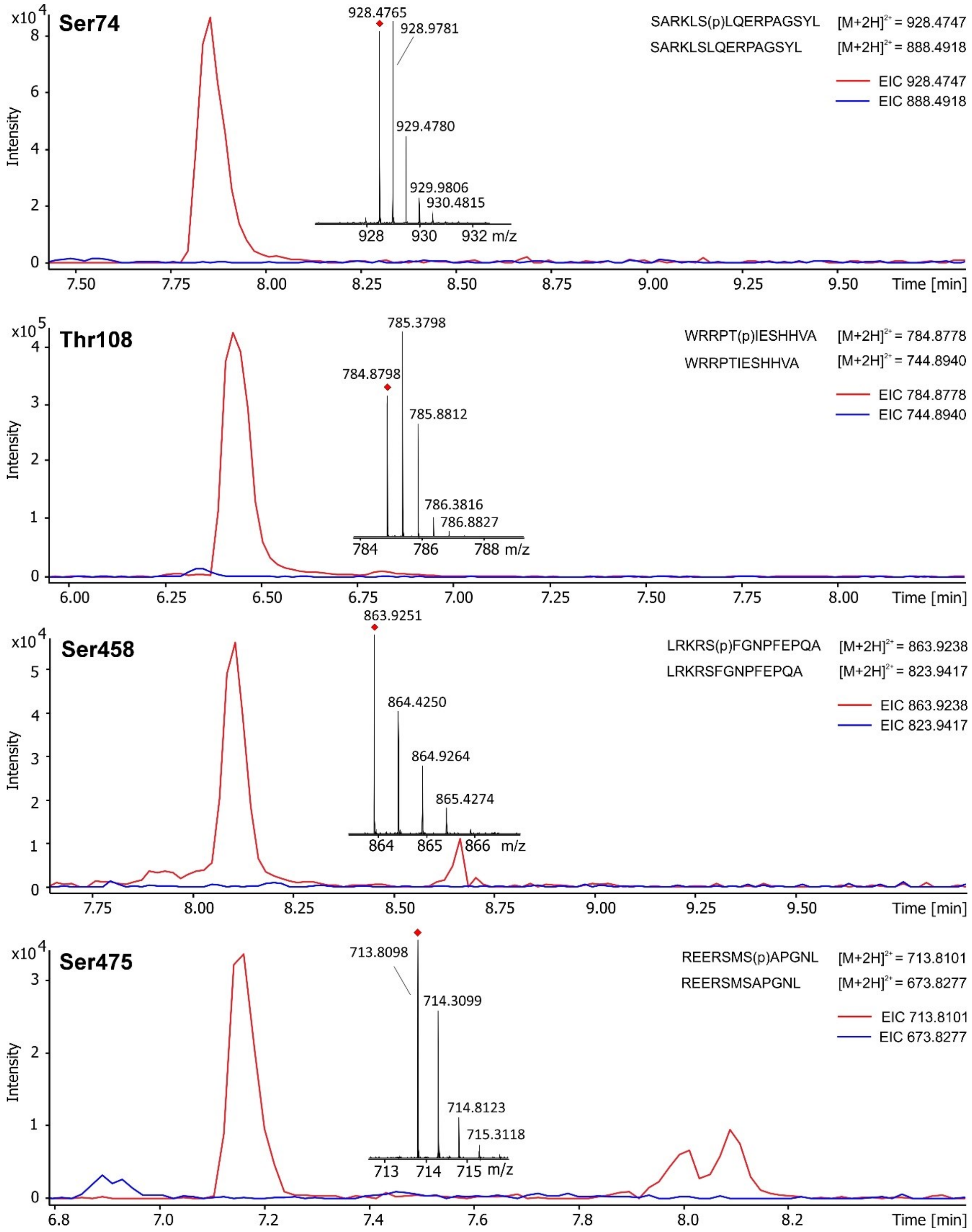

Supplemental Figure S2. Detection of CaMKK1 phosphorylated peptides by FT-ICR mass spectrometry. Extract ion chromatograms (EIC) of phosphorylated peptides are shown in red. The blue lines represent the EIC of the non-phosphorylated forms of the same peptides. The insets show the zoomed, high-resolution MS spectra of phosphorylated peptides. The phosphorylation sites in all peptides were determined based on collision-induced dissociation spectra.

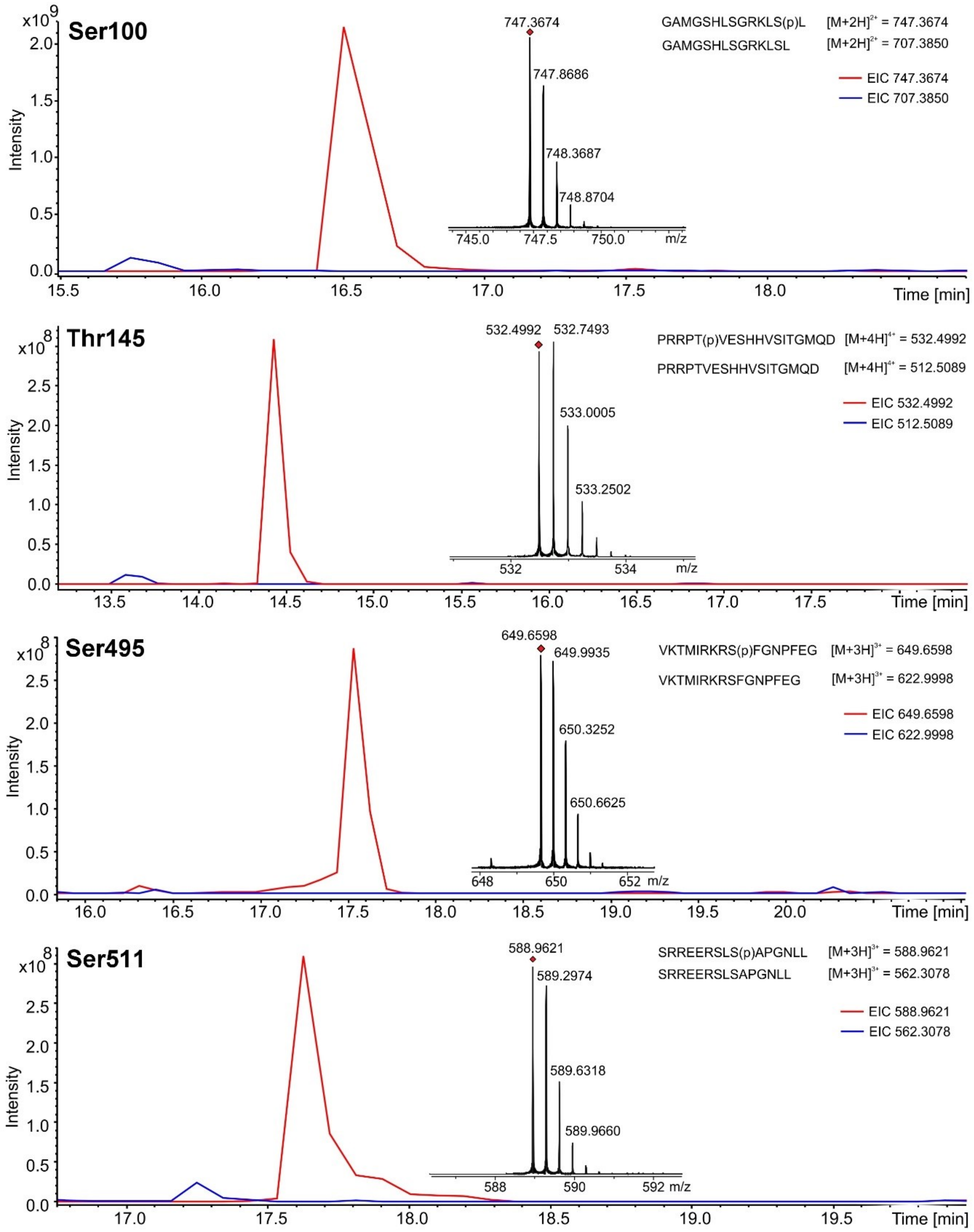

Supplemental Figure S3. Detection of CaMKK2 phosphorylated peptides by FT-ICR mass spectrometry. Extract ion chromatograms (EIC) of phosphorylated peptides are shown in red. The blue lines represent the EIC of the non-phosphorylated forms of the same peptides. The insets show the zoomed, high-resolution MS spectra of phosphorylated peptides. The phosphorylation sites in all peptides were determined based on the collision-induced dissociation spectra.

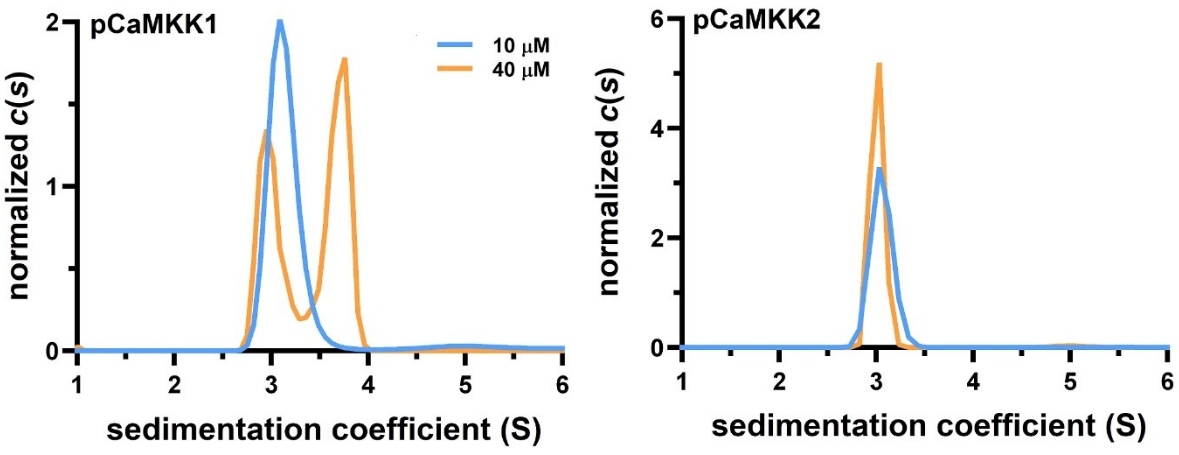

Supplemental Figure S4. Comparison of area-normalized *c*(*s*) distributions of pCaMKK1 and pCaMKK2 at two different concentrations.

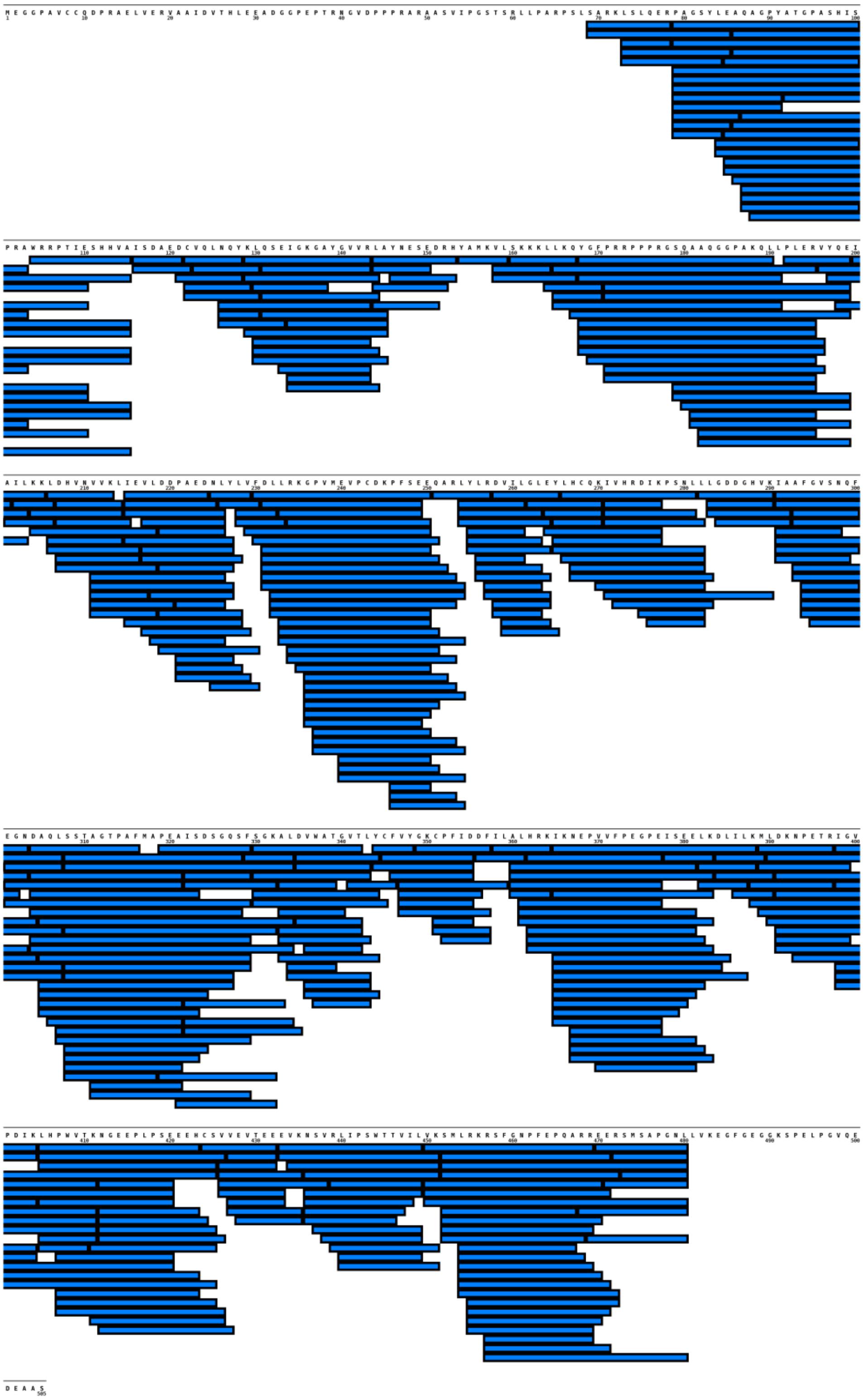

Supplemental Figure S5. Sequence coverage of CaMKK1 (residues 67-480) assessed by HDX. A sequence coverage of 99.5% was obtained for the construct with 414 unique peptides. The map was created using DrawMap script in MSTools (http://peterslab.org/MSTools/).

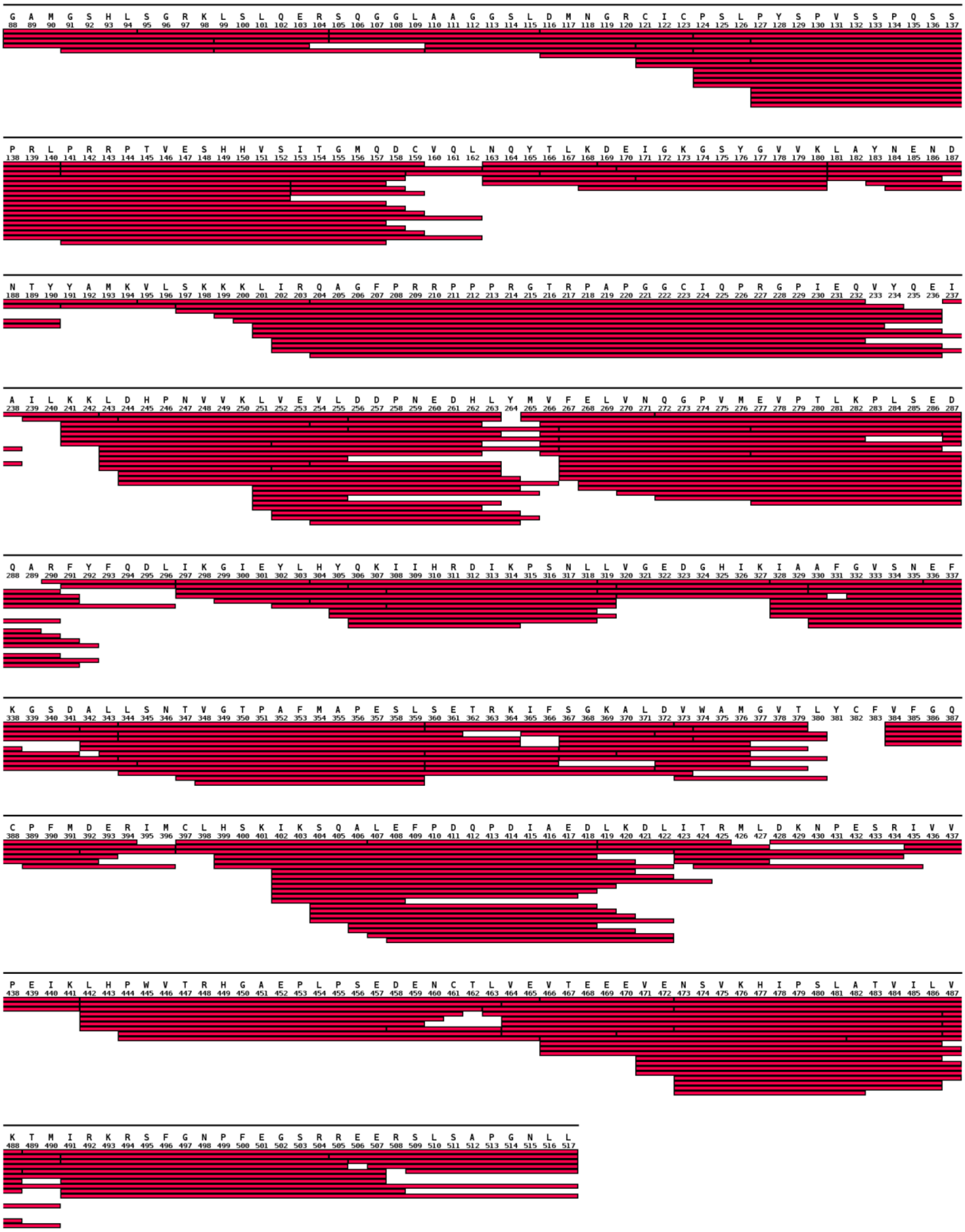

Supplemental Figure S6. Sequence coverage of CaMKK2 (residues 93−517) assessed by HDX. A sequence coverage of 99.3% was obtained for the construct with 282 unique peptides. The map was created using DrawMap script, part of MSTools (http://peterslab.org/MSTools/).

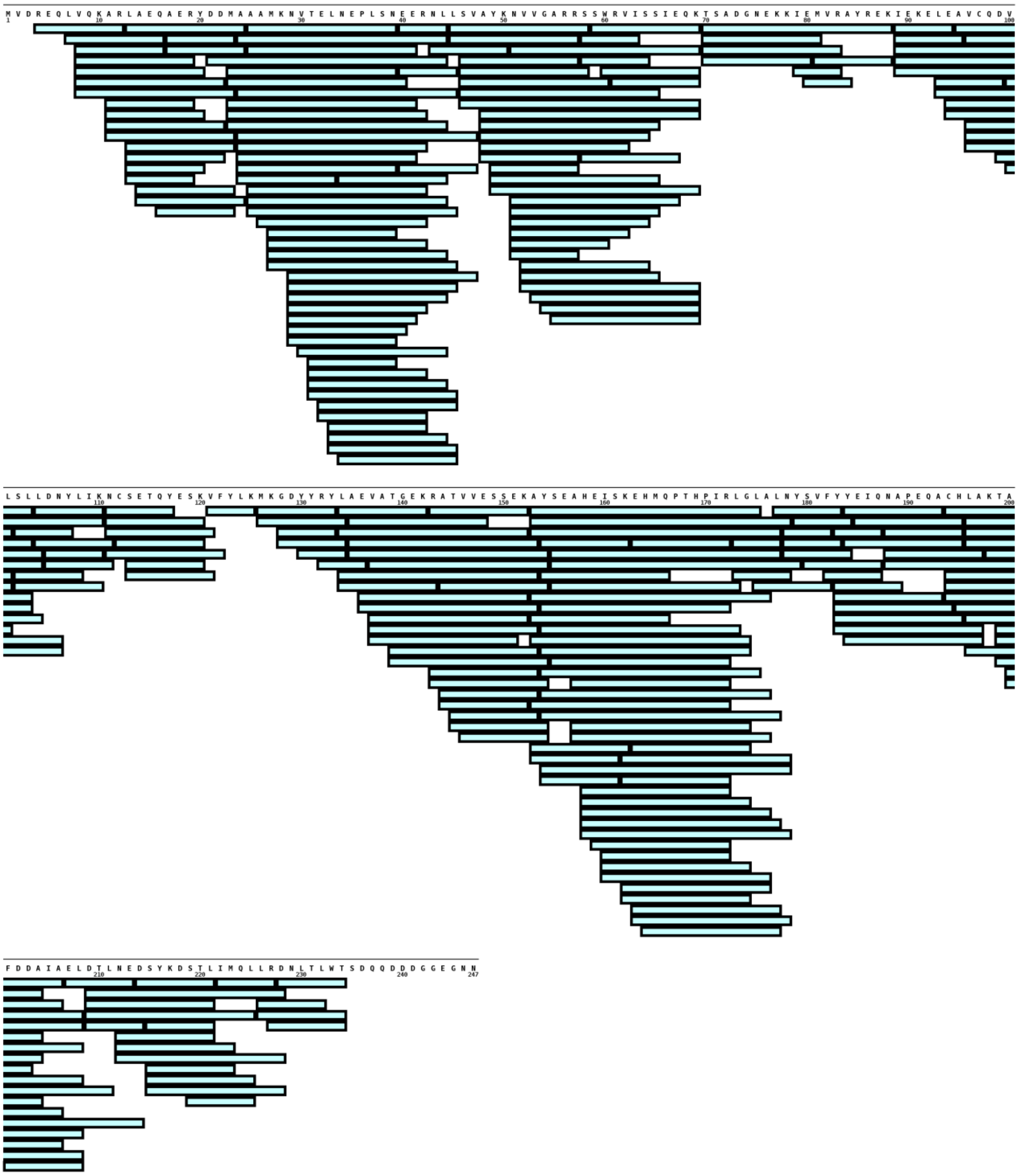

Supplemental Figure S7. Sequence coverage of 14-3-3γ (residues 1−235) assessed by HDX. A sequence coverage of 98.3% was obtained for the construct with 273 unique peptides. The map was created using DrawMap script, part of MSTools (http://peterslab.org/MSTools/).

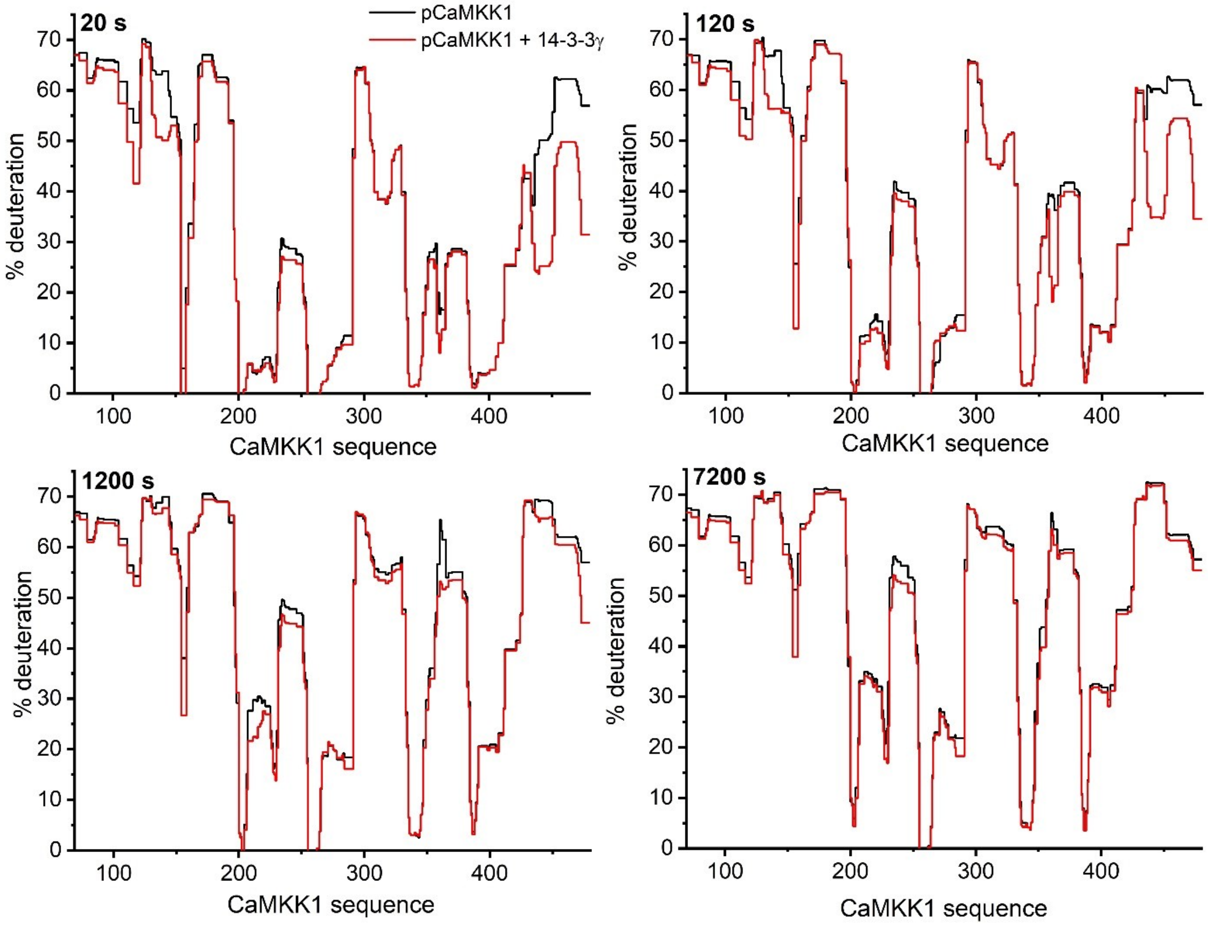

Supplemental Figure S8. Changes in deuterium uptake in pCaMKK1 from binding to 14-3-3γ. Protection plots showing the deuteration levels of pCaMKK1 alone (black) and with 14-3-3γ (red) at four different deuteration times: 20 s, 2 min, 20 min and 2 h.

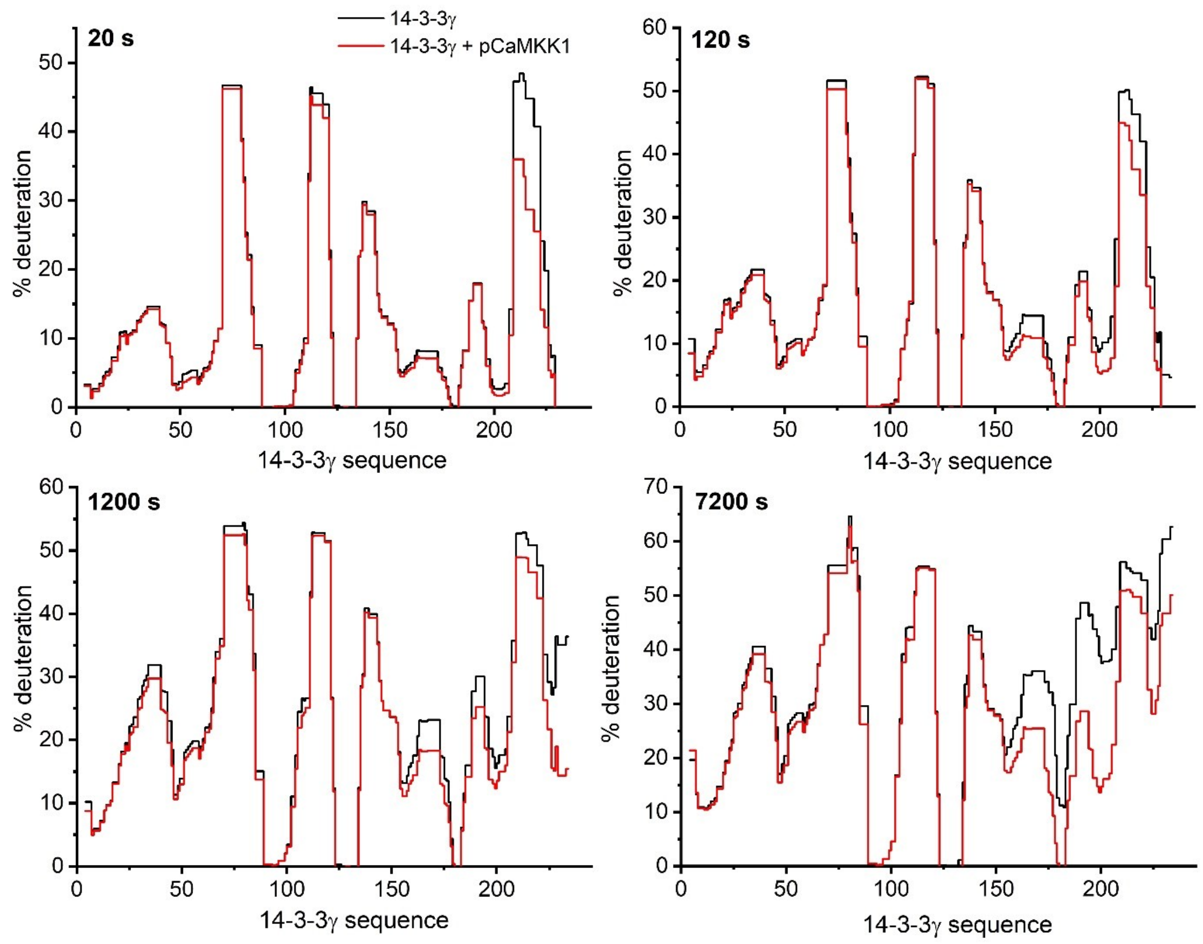

Supplemental Figure S9. Changes in deuterium uptake in 14-3-3γ from binding to pCaMKK1. Protection plots showing the deuteration levels of 14-3-3γ alone (black) and with pCaMKK1 (red) at four different deuteration times: 20 s, 2 min, 20 min and 2 h.

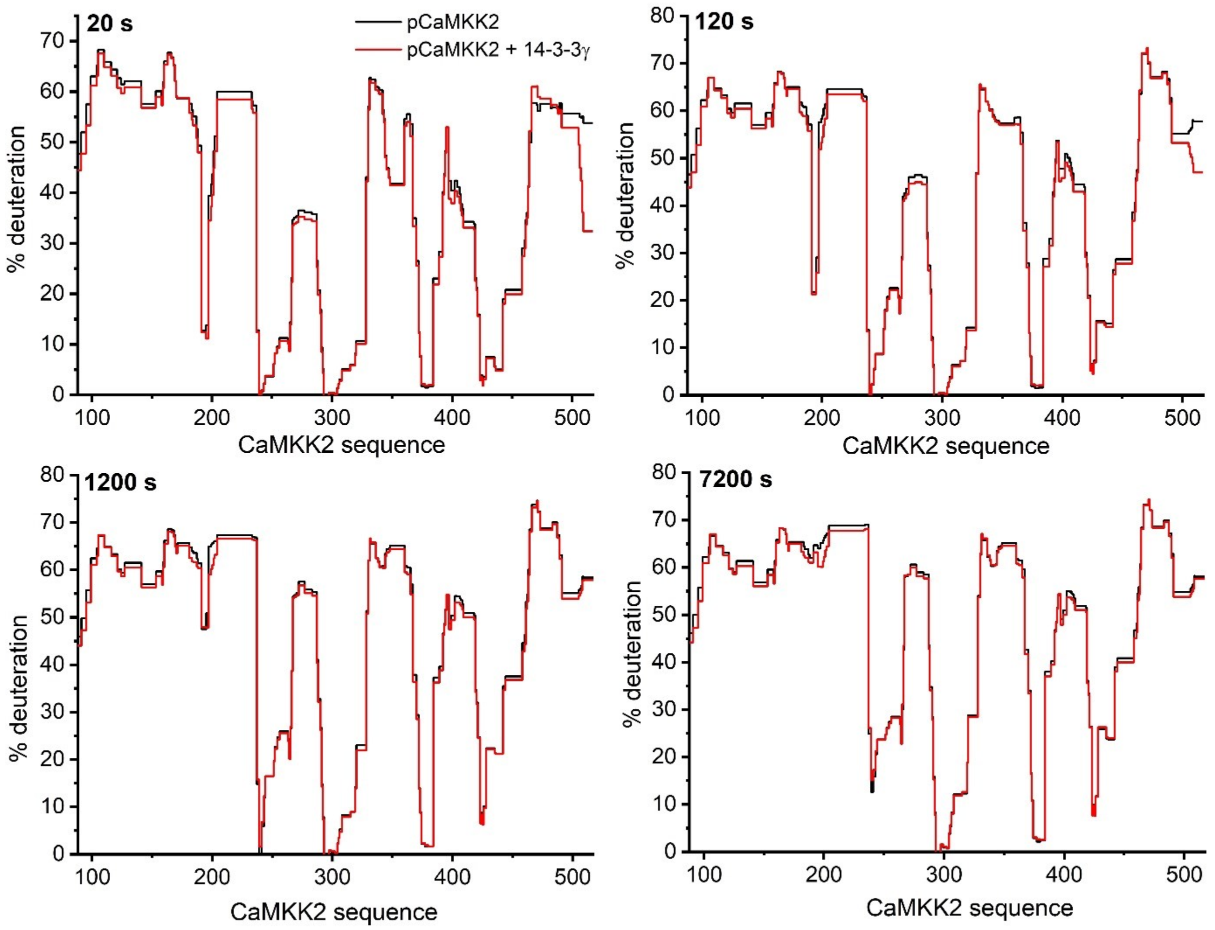

Supplemental Figure S10. Changes in deuterium uptake in pCaMKK2 from binding to 14-3-3γ. Protection plots showing the deuteration levels of pCaMKK2 alone (black) and pCaMKK2 with 14-3-3γ (red) at four different deuteration times: 20 s, 2 min, 20 min and 2 h.

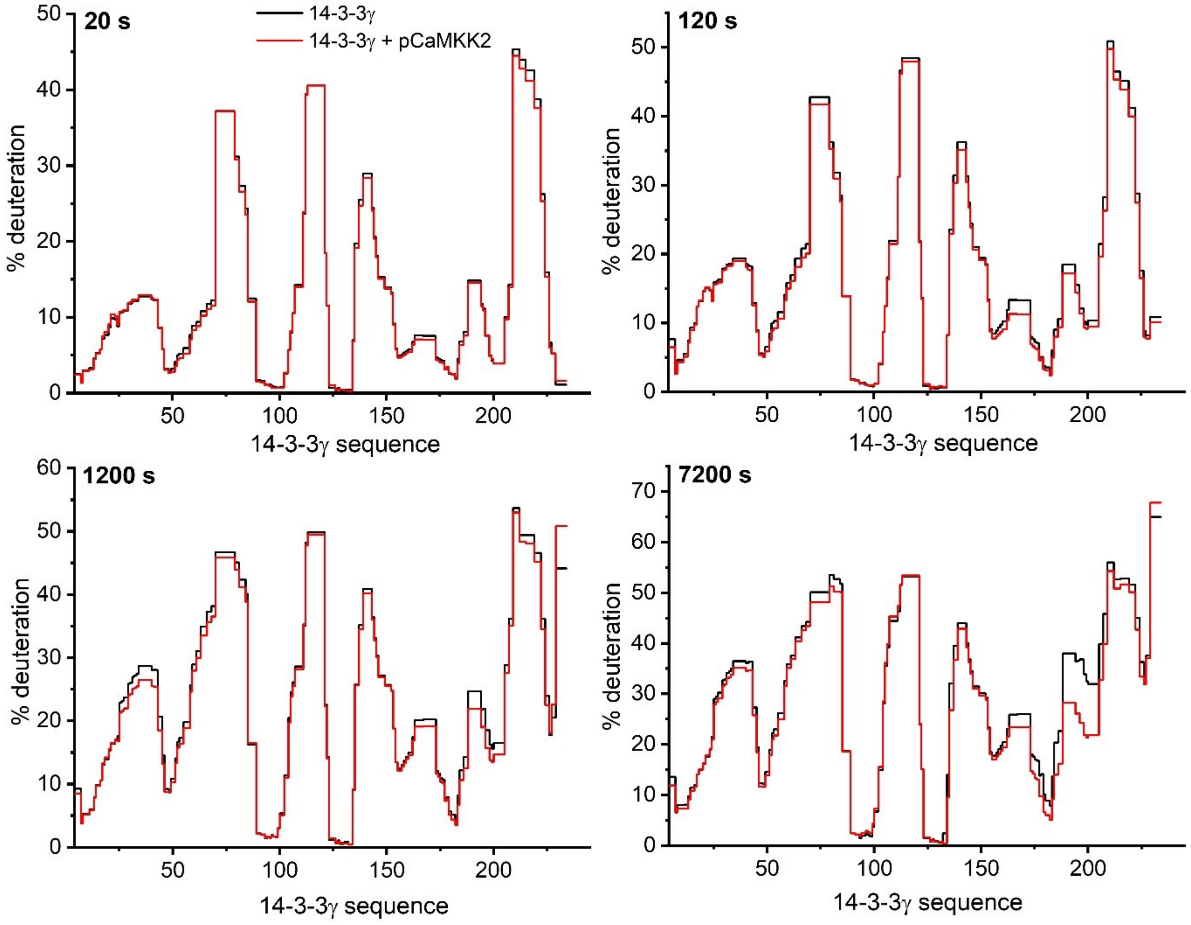

Supplemental Figure S11. Changes in deuterium uptake in 14-3-3γ from binding to pCaMKK2. Protection plots showing the deuteration levels of 14-3-3γ alone (black) and with pCaMKK2 (red) at four different deuteration times: 20 s, 2 min, 20 min and 2 h.

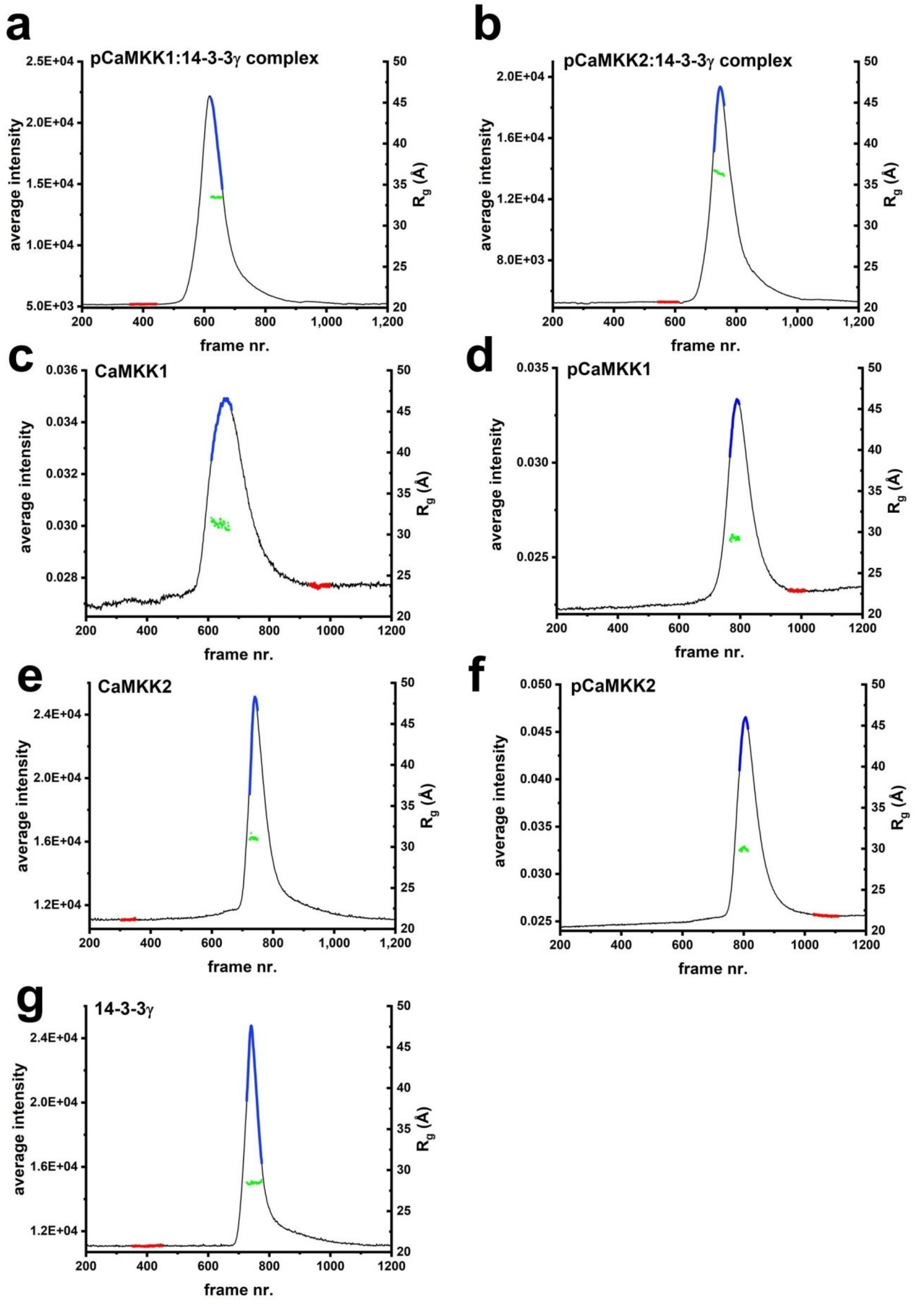

Supplemental Figure S12. SEC-SAXS elution profiles of the pCaMKK1:14-3-3γ complex (a), the pCaMKK2:14-3-3γ complex (b), CaMKK1 (c), pCaMKK1 (d), CaMKK2 (e), pCaMKK2 (f) and 14-3-3γ (g). The regions that were used for further analysis (selected on the basis of constant R_g where possible) are shown in blue. Green dots indicate R_g values for the selected frames. The regions that were used for buffer subtraction are shown in red.

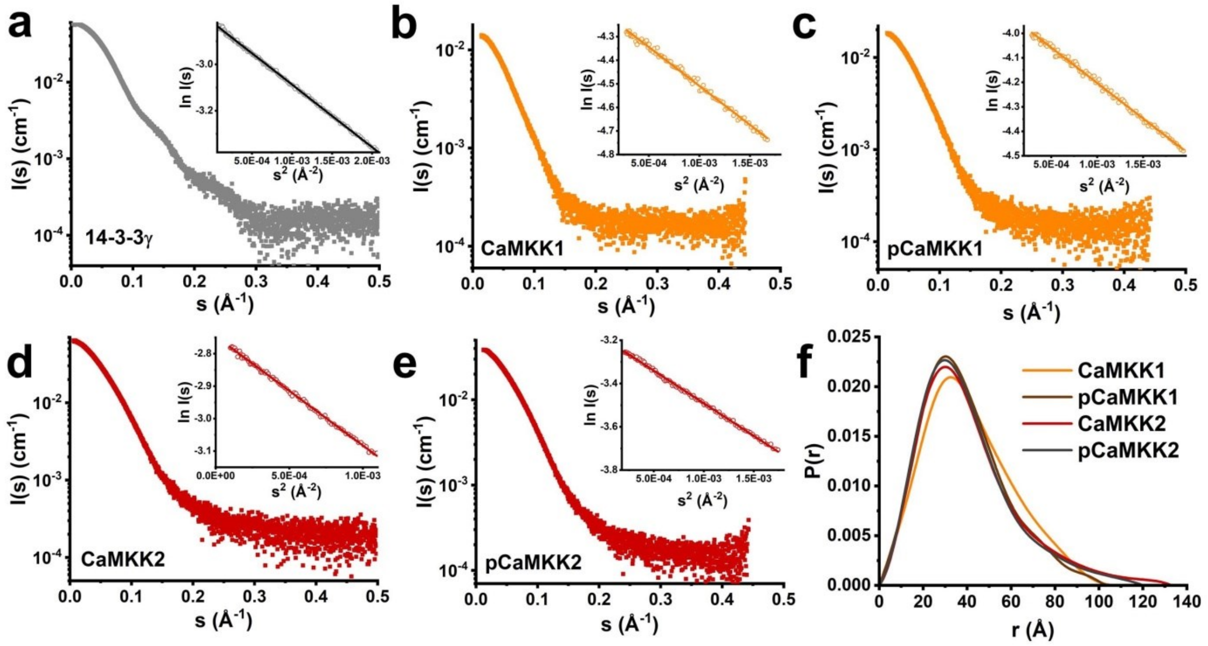

Supplemental Figure S13. Scattering intensity as a function of the scattering vector *s* (s=4πsin(θ/λ), where 2θ is the scattering angle, and λ is the wavelength) of the 14-3-3γ (a), CaMKK1 (b), pCaMKK1 (c), CaMKK2 (d), and pCaMKK2 (e). The insets show Guinier plots of the scattering data. (f) Comparison of $P(r)$ functions of unphosphorylated and phosphorylated CaMKK1 and CaMKK2.

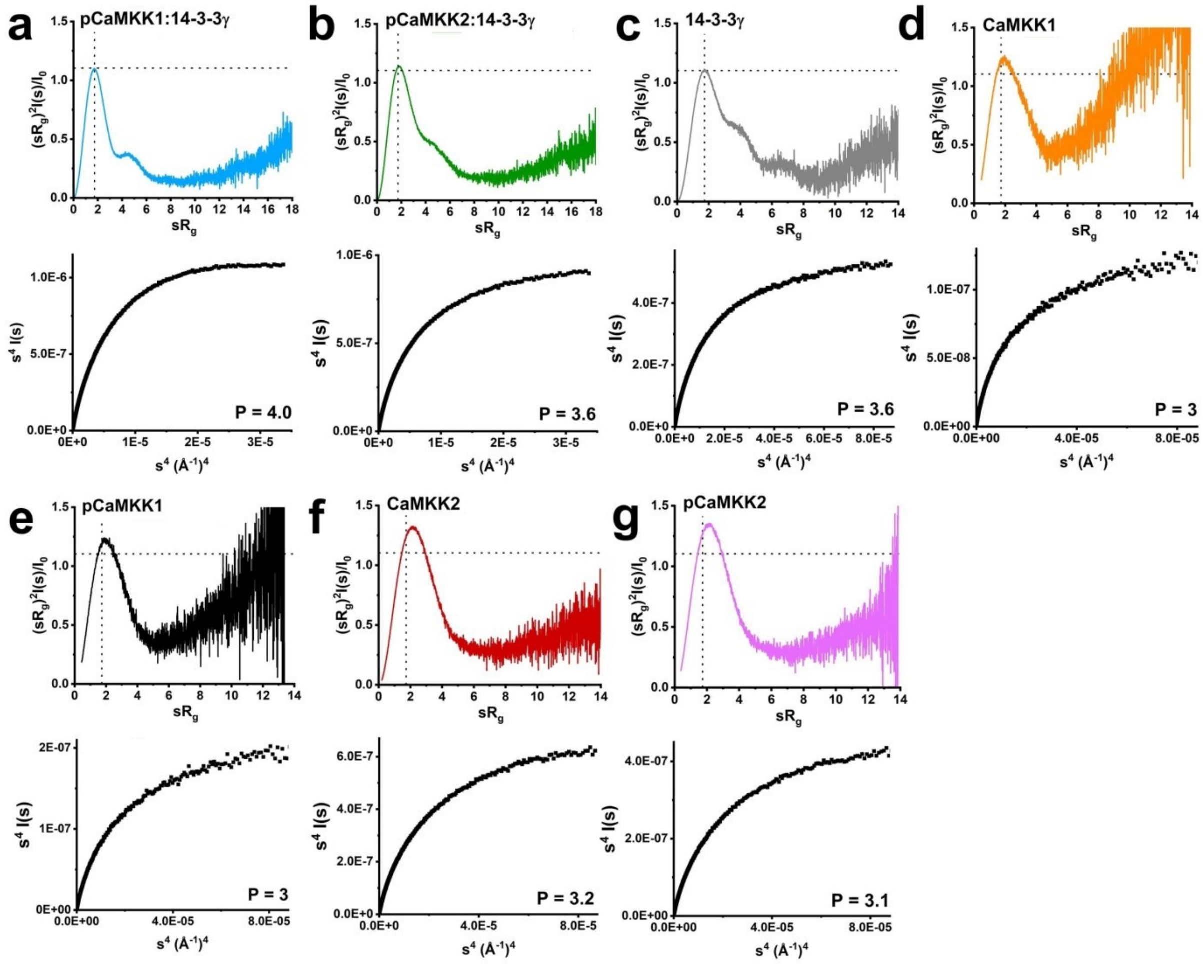

Supplemental Figure S14. Dimensionless Kratky plots ($(sR_g)^2I(s)/I_0$ versus sR_g, where s is the momentum transfer, $I(s)$ is the scattering intensity, and I_0 is the extrapolated intensity at zero angle) and Porod-Debye plots ($s^4I(s)$ vs s^4) for the pCaMKK1:14-3-3γ complex (a), the pCaMKK2:14-3-3γ complex (b), 14-3-3γ (c), CaMKK1 (d), pCaMKK1 (e), CaMKK2 (f), and pCaMKK2 (g). The Porod-Debye exponent values (P) were calculated using the program ScÅtter IV (https://bl1231.als.lbl.gov/scatter/).

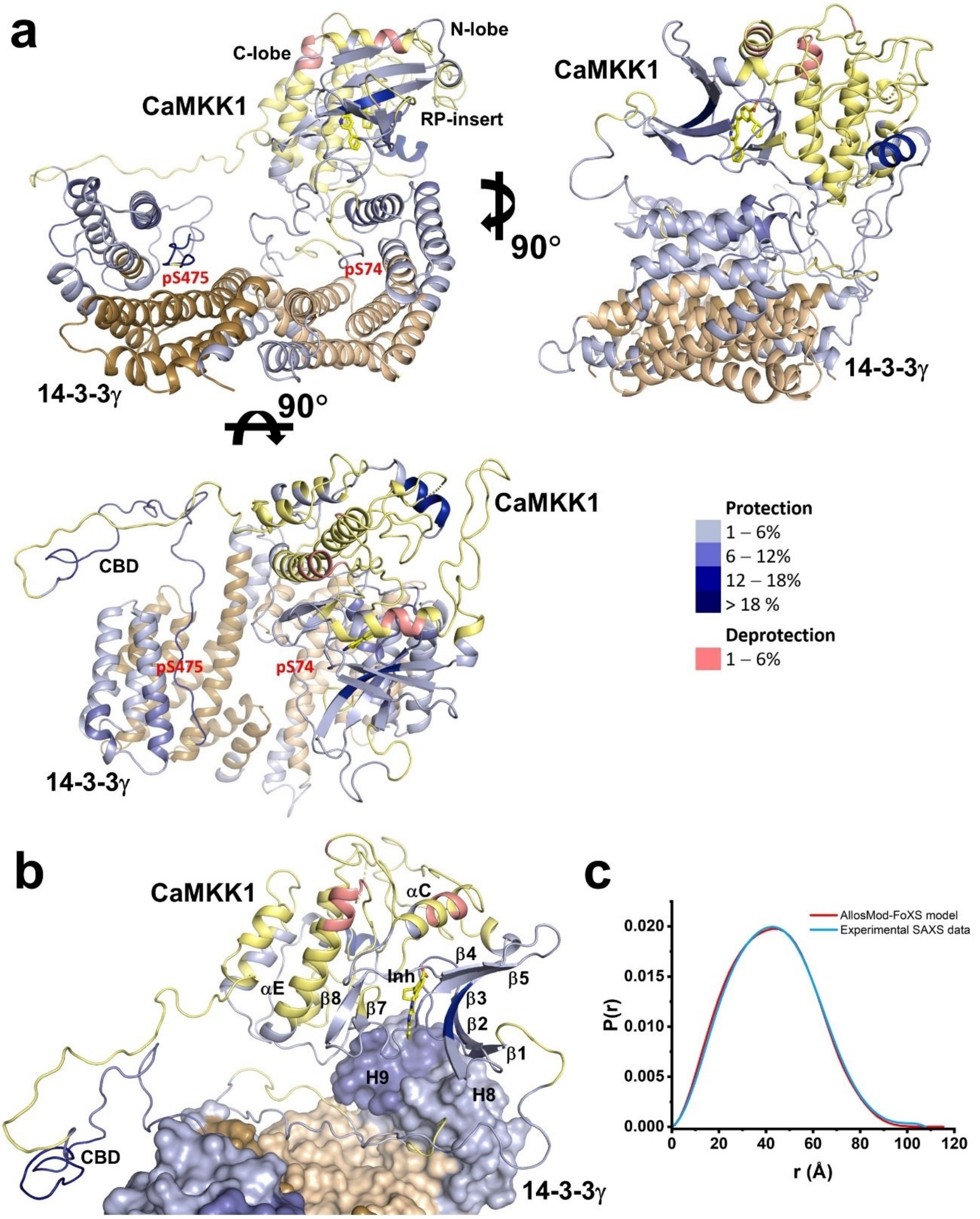

Supplemental Figure S15. SAXS-based structural analysis of the pCaMKK1:14-3-3γ complex. (a) Best scoring AllosMod-FoXS model of the pCaMKK1:14-3-3γ complex. (b) Detailed view of the binding interface between the C-terminal part of 14-3-3γ and the kinase domain of pCaMKK1. The model is colored according to the changes in deuteration kinetics after complex formation for a deuteration time of 120 s. The inhibitor present in the crystal structure of the kinase domain of CaMKK1 (PDB ID: 6CD6) is shown as yellow sticks to indicate the position of the ATP binding site. (c) Comparison of the distance distribution function *P*(*r*) calculated from scattering data of the pCaMKK1:14-3-3γ complex with the calculated *P*(*r*) of the best-scoring AllosMod-FoXS model of the complex (shown in red).

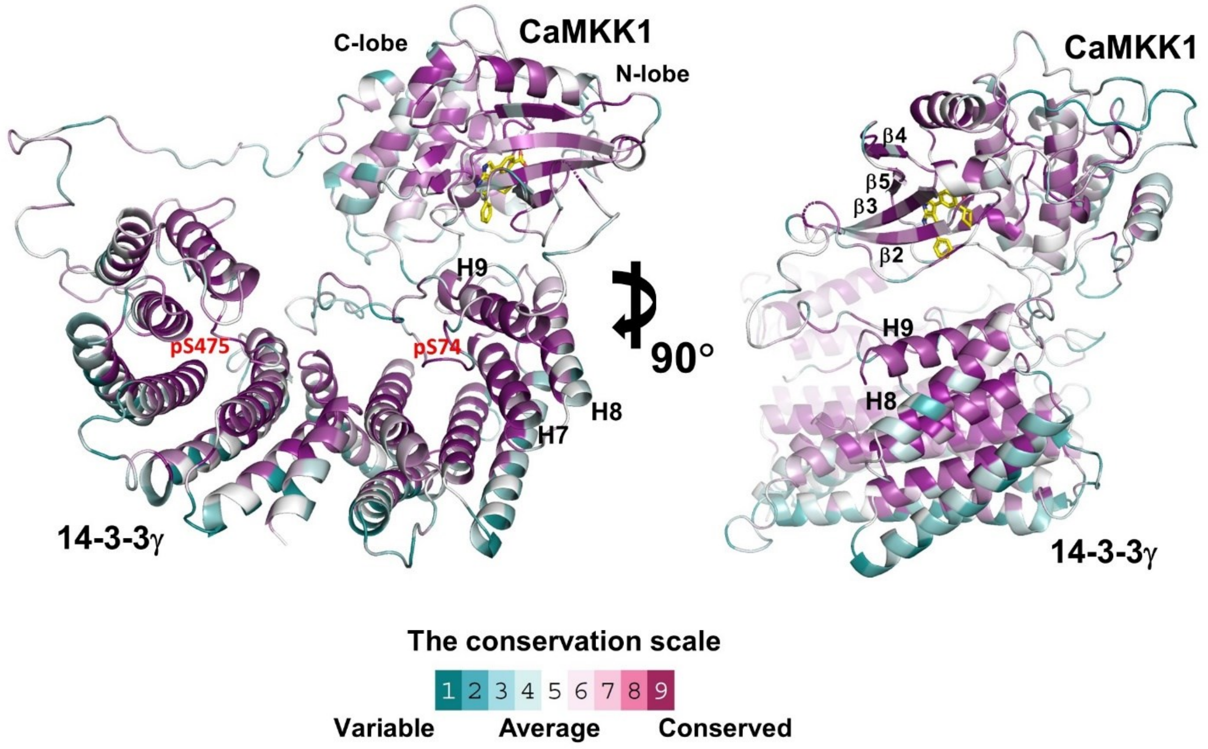

Supplemental Figure S16. Sequence conservation analysis of residues forming the binding interface between pCaMKK1 KD and 14-3-3γ. Sequence conservation was calculated using the ConSurf server (https://consurf.tau.ac.il/consurf_index.php) (14).

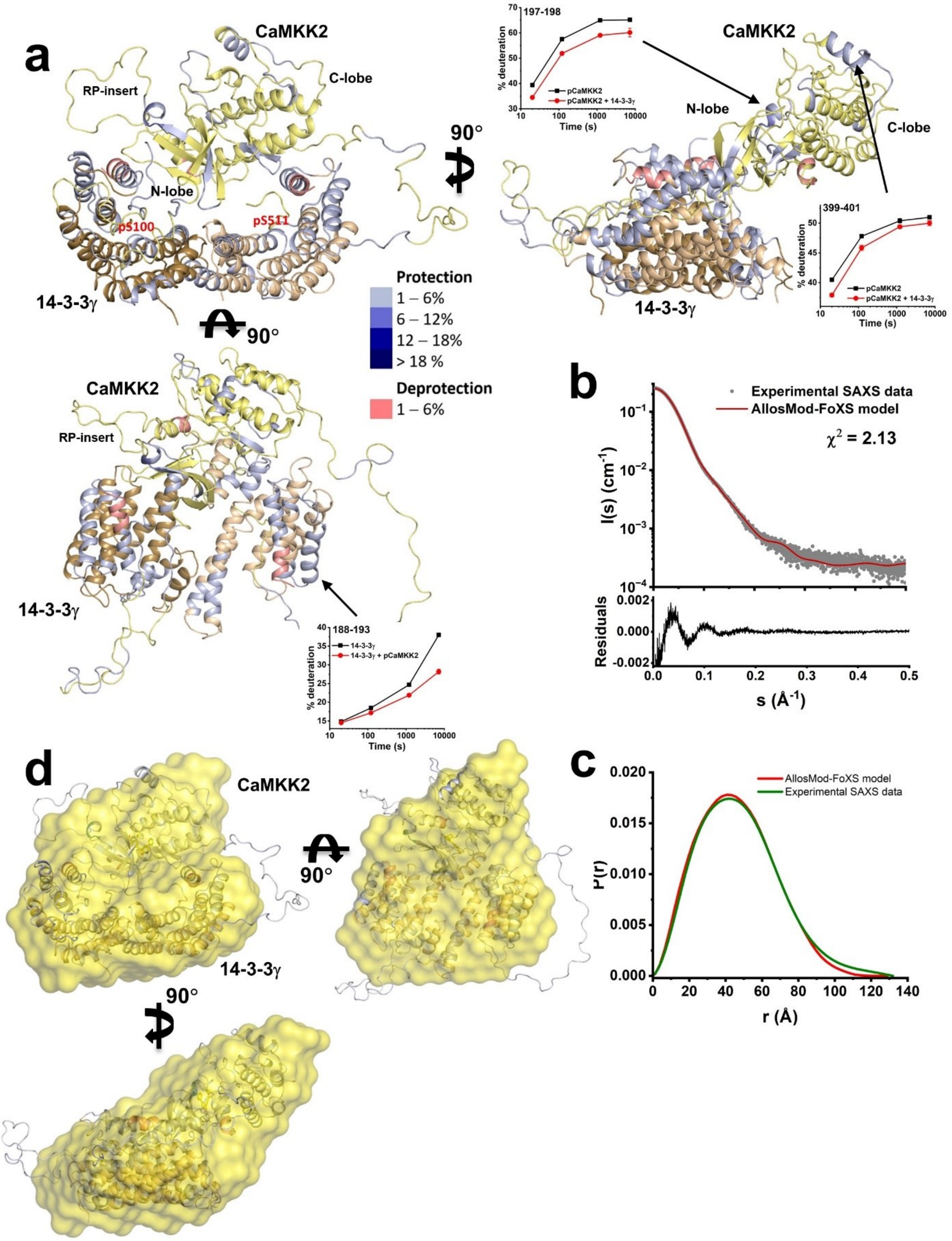

Supplemental Figure S17. SAXS-based structural analysis of the pCaMKK2:14-3-3γ complex. (a) The best-scoring AllosMod-FoXS model of the pCaMKK2:14-3-3γ complex calculated using and the crystal structures of the kinase domain of CaMKK2 (PDB ID: 5UY6) and 14-3-3γ with bound CaMKK phosphopeptides (PDB ID: 6FEL and 6EWW (15)). The inhibitor present in the crystal structure of the kinase domain indicates the position of the ATP

binding site (shown as yellow sticks). The model is colored according to changes in deuterium uptake after complex formation for a deuteration time of 20 min. Graphs show representative HDX kinetics for selected pCaMKK2 and 14-3-3γ regions with changed deuterium exchange kinetics after complex formation. Deuterium exchange is expressed as percentage of the maximum theoretical deuteration level of pCaMKK2 or 14-3-3γ alone (black squares) and in the complex (red circles). (b) Experimental scattering curve of the pCaMKK2:14-3-3γ complex superimposed with the calculated curve of the best-scoring AllosMod-FoXS model of the complex (shown in red). (c) Comparison of the distance distribution function *P*(*r*) calculated from scattering data of the pCaMKK2:14-3-3γ complex with the calculated *P*(*r*) of the best-scoring AllosMod-FoXS model of the complex (shown in red). (d) Averaged and filtered ab initio molecular envelope of the pCaMKK2:14-3-3γ complex (shown in yellow) calculated from SAXS data with a superimposed AllosMod-FoXS model of the complex. Ab initio shapes from fifteen iterations of DAMMIF (8) were averaged and filtered using the DAMAVER package (16).

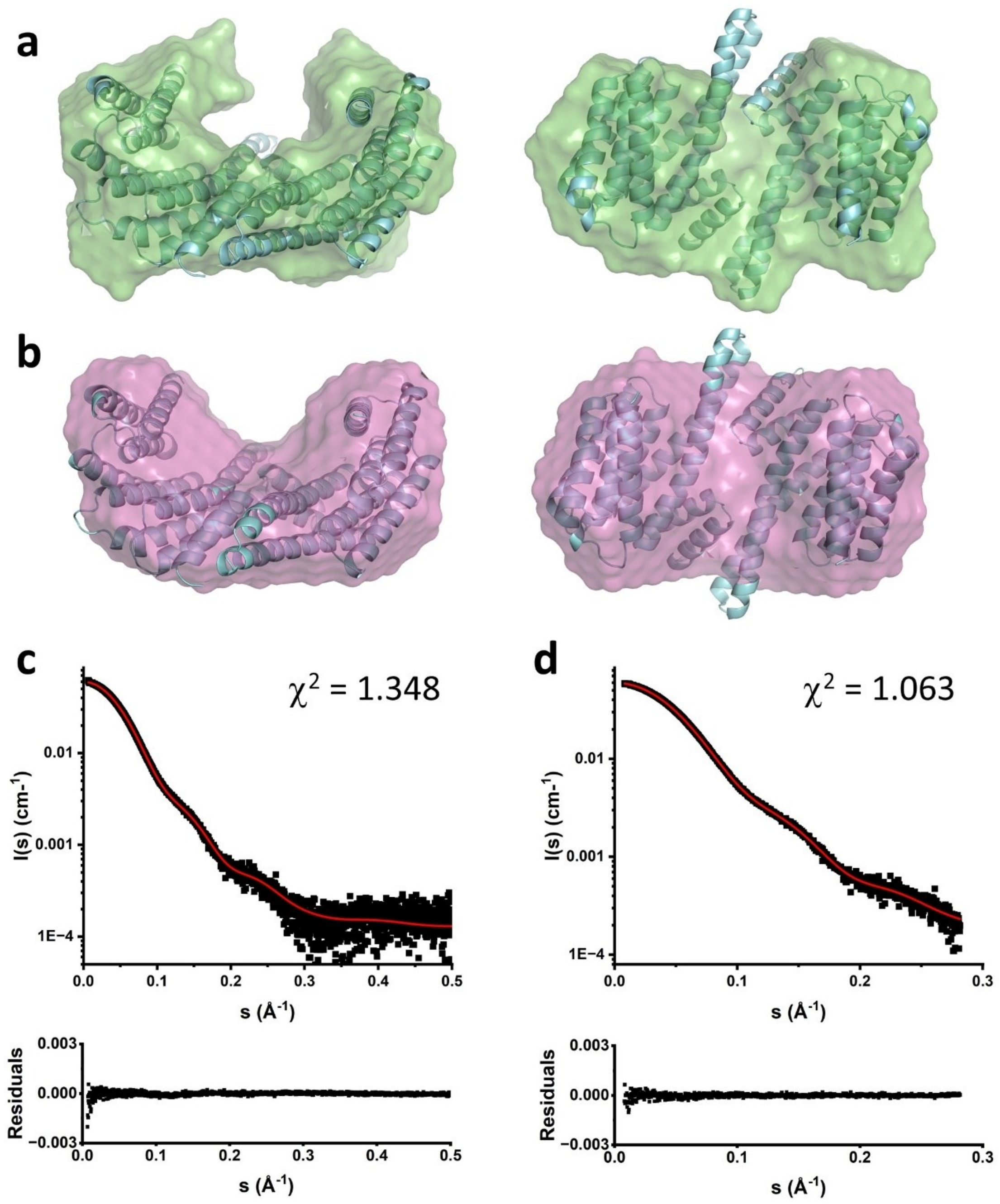

Supplemental Figure S18. Comparison of the crystal structure of 14-3-3γ protein with scattering data. (a) Ab initio molecular envelope of 14-3-3γ calculated from SAXS data with superposed crystal structure of 14-3-3γ (PDB ID: 2B05). Ab initio shapes from fifteen iterations of DAMMIF (8) were averaged and filtered using the DAMAVER package (16), and the averaged and filtered envelope was refined using one run of DAMMIN (17). (b) Averaged and filtered ab initio molecular envelope of 14-3-3γ with superposed crystal structure of 14-3-3γ. (c) Comparison of the calculated scattering curve of the 14-3-3γ crystal structure (red line) with experimental scattering data. (d) Comparison of the experimental SAXS profile of 14-3-3γ with the fit of its DAMMIN ab initio model (red line).

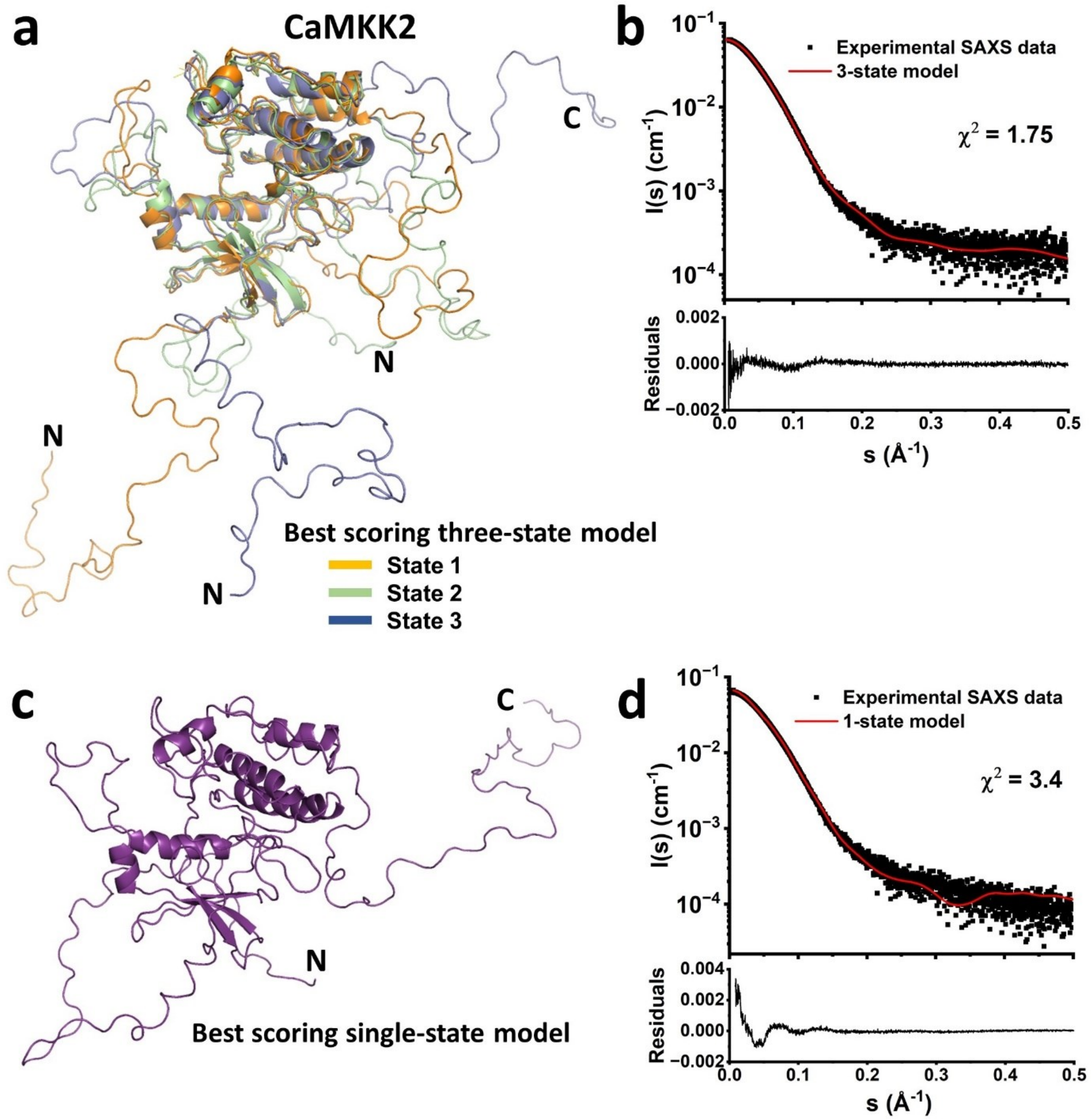

Supplemental Figure S19. Comparison of multi- and single-state models of CaMKK2. (a) The best scoring three-state model of CaMKK2 contains one compact state (State 2, R_g = 28.8 Å) with a population weight of 45% and two extended states with population weights of 29% (State 1, R_g = 37.1 Å) and 26% (State 3, R_g = 34.4 Å). (b) Comparison of the experimental SAXS profile of CaMKK2 with the fit of its best-scoring three-state model (red line). (c) The best-scoring single-state model of CaMKK2 (R_g = 40 Å). (d) Comparison of the experimental SAXS profile of CaMKK2 with the fit of its best-scoring single-state model (red line).

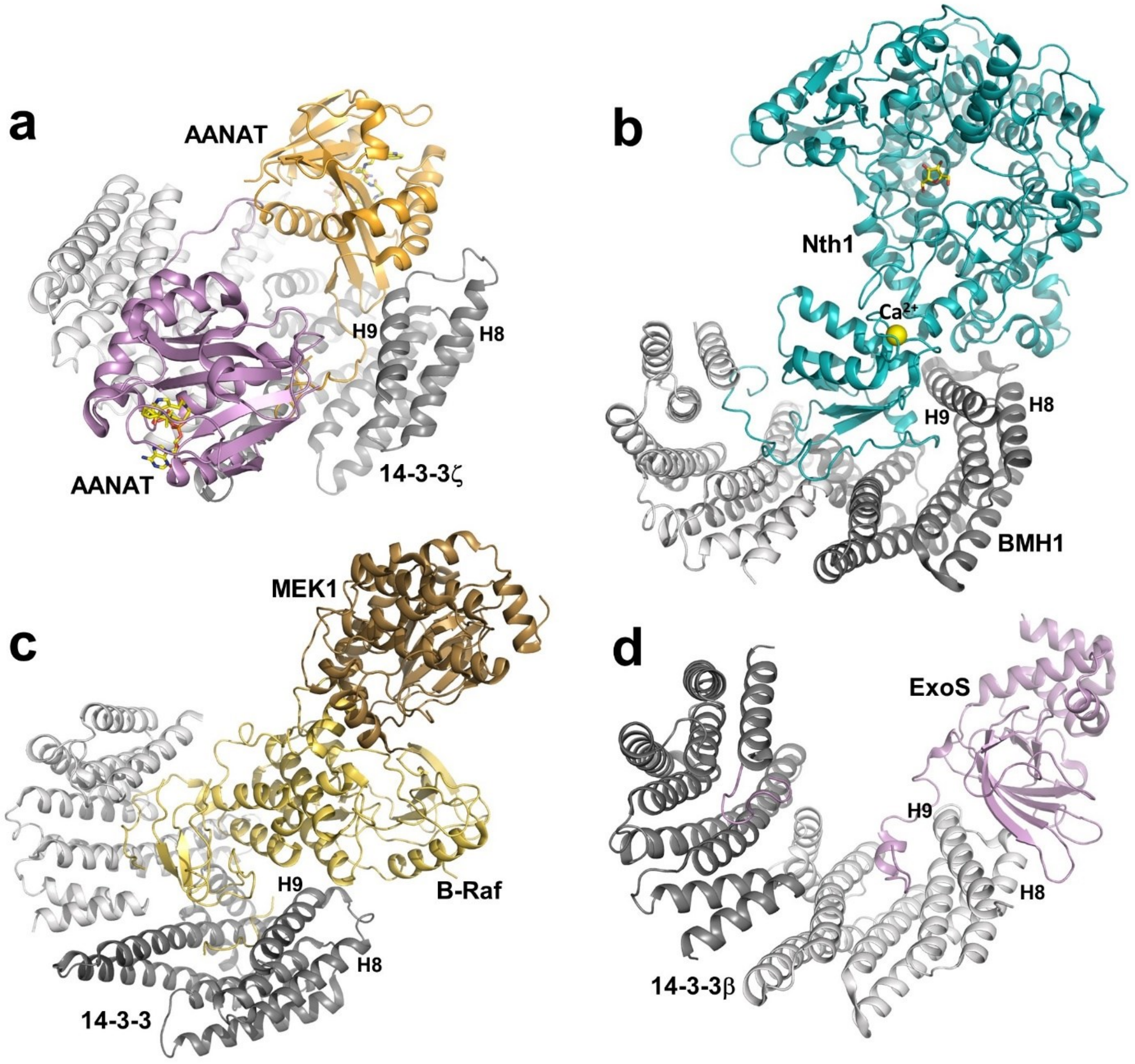

Supplemental Figure S20. The last two C-terminal helices of 14-3-3 proteins are often involved in interactions with target proteins. (a) Crystal structure of the AANAT:14-3-3ζ complex (PDB: 1IB1 (18)). The bisubstrate analog in shown as sticks. (b) Crystal structure of the Nth1:14-3-3 complex (PDB: 5N6N (19)). The sucrose bound in the active site is shown as sticks. Ca^{2+} ion is shown as orange sphere. (c) Autoinhibited B-RAF:MEK1:14-3-3 complex (PDB ID: 6NYB (20)). (d) Crystal structures of the ExoS:14-3-3β complex (PDB ID: 6GN8 (21)).

Supplemental References

1. Schuck P (2000) Size-distribution analysis of macromolecules by sedimentation velocity ultracentrifugation and lamm equation modeling. Biophys J 78:1606-1619.
2. Houtman JC, Brown PH, Bowden B, Yamaguchi H, Appella E, Samelson LE, Schuck P (2007) Studying multisite binary and ternary protein interactions by global analysis of isothermal titration calorimetry data in SEDPHAT: application to adaptor protein complexes in cell signaling. Protein Sci 16:30-42.
3. Vecer J, Herman P (2011) Maximum Entropy Analysis of Analytically Simulated Complex Fluorescence Decays. Journal of Fluorescence 21:873-881.
4. Bryan RK (1990) Maximum-Entropy Analysis of Oversampled Data Problems. European Biophysics Journal 18:165-174.
5. Franke D, Kikhney AG, Svergun DI (2012) Automated acquisition and analysis of small angle X-ray scattering data. Nucl Instrum Meth A 689:52-59.
6. Manalastas-Cantos K, Konarev PV, Hajizadeh NR, Kikhney AG, Petoukhov MV, Molodenskiy DS, Panjkovich A, Mertens HDT, Gruzinov A, Borges C, Jeffries CM, Svergun DI, Franke D (2021) ATSAS 3.0: expanded functionality and new tools for small-angle scattering data analysis. J Appl Crystallogr 54:343-355.
7. de Oliveira Neto M, de Freitas Fernandes A, Piiadov V, Craievich AF, de Araujo EA, Polikarpov I (2022) SAXSMoW 3.0: New advances in the determination of the molecular weight of proteins in dilute solutions from SAXS intensity data on a relative scale. Protein Sci 31:251-258.
8. Franke D, Svergun DI (2009) DAMMIF, a program for rapid ab-initio shape determination in small-angle scattering. Journal of Applied Crystallography 42:342-346.
9. Petoukhov MV, Franke D, Shkumatov AV, Tria G, Kikhney AG, Gajda M, Gorba C, Mertens HD, Konarev PV, Svergun DI (2012) New developments in the program package for small-angle scattering data analysis. J Appl Crystallogr 45:342-350.
10. Weinkam P, Pons J, Sali A (2012) Structure-based model of allostery predicts coupling between distant sites. Proc Natl Acad Sci U S A 109:4875-4880.
11. Schneidman-Duhovny D, Hammel M, Tainer JA, Sali A (2016) FoXS, FoXSDock and MultiFoXS: Single-state and multi-state structural modeling of proteins and their complexes based on SAXS profiles. Nucleic Acids Res 44:W424-429.
12. Ortega A, Amoros D, Garcia de la Torre J (2011) Prediction of hydrodynamic and other solution properties of rigid proteins from atomic- and residue-level models. Biophys J 101:892-898.
13. Emsley P, Cowtan K (2004) Coot: model-building tools for molecular graphics. Acta Crystallogr D Biol Crystallogr 60:2126-2132.
14. Ashkenazy H, Abadi S, Martz E, Chay O, Mayrose I, Pupko T, Ben-Tal N (2016) ConSurf 2016: an improved methodology to estimate and visualize evolutionary conservation in macromolecules. Nucleic Acids Res 44:W344-350.
15. Psenakova K, Petrvalska O, Kylarova S, Lentini Santo D, Kalabova D, Herman P, Obsilova V, Obsil T (2018) 14-3-3 protein directly interacts with the kinase domain of calcium/calmodulin-dependent protein kinase kinase (CaMKK2). Biochimica et biophysica acta General subjects 1862:1612-1625.
16. Volkov VV, Svergun DI (2003) Uniqueness of ab initio shape determination in small-angle scattering. Journal of Applied Crystallography 36:860-864.
17. Svergun DI (1999) Restoring low resolution structure of biological macromolecules from solution scattering using simulated annealing. Biophys J 76:2879-2886.
18. Obsil T, Ghirlando R, Klein DC, Ganguly S, Dyda F (2001) Crystal structure of the 14-3-3zeta:serotonin N-acetyltransferase complex. a role for scaffolding in enzyme regulation. Cell 105:257-267.
19. Alblova M, Smidova A, Docekal V, Vesely J, Herman P, Obsilova V, Obsil T (2017) Molecular basis of the 14-3-3 protein-dependent activation of yeast neutral trehalase Nth1. Proc Natl Acad Sci U S A 114:E9811-E9820.
20. Park E, Rawson S, Li K, Kim BW, Ficarro SB, Pino GG, Sharif H, Marto JA, Jeon H, Eck MJ (2019) Architecture of autoinhibited and active BRAF-MEK1-14-3-3 complexes. Nature 575:545-550.
21. Karlberg T, Hornyak P, Pinto AF, Milanova S, Ebrahimi M, Lindberg M, Pullen N, Nordstrom A, Loverli E, Caraballo R, Wong EV, Nareoja K, Thorsell AG, Elofsson M, De La Cruz EM,

Bjorkegren C, Schuler H (2018) 14-3-3 proteins activate Pseudomonas exotoxins-S and -T by chaperoning a hydrophobic surface. Nature communications 9:3785.
